# Unraveling 50-Year-Old Clues Linking Neurodegeneration and Cancer to Cycad Toxins: Are microRNAs Common Mediators?

**DOI:** 10.3389/fgene.2012.00192

**Published:** 2012-09-28

**Authors:** Peter Spencer, Rebecca C. Fry, Glen E. Kisby

**Affiliations:** ^1^Global Health Center, Oregon Health and Science UniversityPortland, OR, USA; ^2^Gillings School of Global Public Health, University of North CarolinaChapel Hill, NC, USA; ^3^Basic Medical Sciences, College of Osteopathic Medicine of the Pacific Northwest, Western University of Health SciencesLebanon, OR, USA

**Keywords:** ALS-PDC, Alzheimer disease, amyotrophic lateral sclerosis, methylazoxymethanol, BMAA, formaldehyde, DNA damage, colon cancer

## Abstract

Recognition of overlapping molecular signaling activated by a chemical trigger of cancer and neurodegeneration is new, but the path to this discovery has been long and potholed. Six conferences (1962–1972) examined the puzzling neurotoxic and carcinogenic properties of a then-novel toxin [cycasin: methylazoxymethanol (MAM)-β-d-glucoside] in cycad plants used traditionally for food and medicine on Guam where a complex neurodegenerative disease plagued the indigenous population. Affected families showed combinations of amyotrophic lateral sclerosis (ALS), parkinsonism (P), and/or a dementia (D) akin to Alzheimer’s disease (AD). Modernization saw declining disease rates on Guam and remarkable changes in clinical phenotype (ALS was replaced by P-D and then by D) and in two genetically distinct ALS-PDC-affected populations (Kii-Japan, West Papua-Indonesia) that used cycad seed medicinally. MAM forms DNA lesions – repaired by *O*^6^-methylguanine methyltransferase (MGMT) – that perturb mouse brain development and induce malignant tumors in peripheral organs. The brains of young adult MGMT-deficient mice given a single dose of MAM show DNA lesion-linked changes in cell-signaling pathways associated with miRNA-1, which is implicated in colon, liver, and prostate cancers, and in neurological disease, notably AD. MAM is metabolized to formaldehyde, a human carcinogen. Formaldehyde-responsive miRNAs predicted to modulate MAM-associated genes in the brains of MGMT-deficient mice include miR-17-5p and miR-18d, which regulate genes involved in tumor suppression, DNA repair, amyloid deposition, and neurotransmission. These findings marry cycad-associated ALS-PDC with colon, liver, and prostate cancer; they also add to evidence linking changes in microRNA status both to ALS, AD, and parkinsonism, and to cancer initiation and progression.

## Introduction

Non-coding RNAs in the form of microRNAs (miRNAs) form a class of hundreds of short, non-coding RNA regulatory molecules that base pair with multiple target mRNAs to down-regulate gene expression by mechanisms ranging from translational inhibition to mRNA degradation (O’Carroll and Schaefer, 2012). miRNAs arise from long RNA hairpin transcripts that are processed in the cell nucleus by the Drosha microprocessor complex and then, in the cytoplasm, by Dicer, an endoribonuclease in the RNase III family. One of the two complementary short RNA molecules so formed is integrated into the RISC complex, a ribonucleoprotein complex containing members of the Argonaute (Ago) family of endonucleases, the activity of which is directed against mRNA strands that are complementary to their bound miRNA fragment. After integration into the active RISC complex, miRNAs bind to imperfect complementary sites within the 3′ untranslated regions (UTRs) of their mRNA targets and thereby effect repression of gene expression.

MicroRNAs are heavily represented in the nervous system where they take part in most if not all cell processes, including during development and in adult health and disease. This method of post-transcriptional gene modulation plays an important role in nerve cell differentiation and neuronal function (Vo et al., 2005; Schratt et al., 2006; Leucht et al., 2008; Yu et al., 2008; Siegel et al., 2009; Yoo et al., 2009; Feng and Feng, 2011; Schouten et al., 2012), and changes in expression of specific miRNAs have been found in neurodegenerative diseases (Cogswell et al., 2008; Packer et al., 2008; Haramati et al., 2010; Gascon and Gao, 2012). The functional relevance of miRNAs in brain disease is illustrated by miR-134, activity of which is upregulated in the brain of animals with *status epilepticus* and, when silenced, reduces hippocampal dendritic spine density and makes mice refractory to seizures and resulting hippocampal damage (Jimenez-Mateos et al., 2012). miRNAs are also important, but perhaps not essential, for tumor growth and survival, in that *Dicer1*-deficient sarcoma cells lacking miRNAs are tumorigenic (Ravi et al., 2012).

MicroRNAs have elicited considerable interest among those seeking to understand the pathogenesis, diagnosis, and treatment of key neurodegenerative disorders, notably Alzheimer’s disease (AD), Parkinson’s disease (PD), and amyotrophic lateral sclerosis (ALS). However, the sum total of publications on these subjects (∼125) pales in comparison with those associated with cancer (∼6700), including colon cancer (∼300), the specific subject that links cancer with neurodegenerative disease in this review. The neurodegenerative disease addressed here, namely western Pacific ALS-parkinsonism-dementia complex (ALS-PDC), combines in single patients the clinical and neuropathological features of the key neurodegenerative disorders ALS, atypical parkinsonism, and AD (illustrated videographically at: http://vimeo.com/1621281). While there are several types of dominantly inherited cases of ALS, PD, and AD with specific gene mutations, these represent a minority (∼10%) since most of these disorders occur sporadically, indicating the operation of environmental factors in the presence or absence of genetic susceptibilities. Evidence indicates that the etiology of ALS-PDC is predominantly, if not exclusively, environmental in origin. In particular, the disease has been repeatedly associated with early life exposure via traditional food and/or medicine to naturally occurring chemical constituents of cycad plants (*Cycas* spp.; see: http://vimeo.com/1621281), including methylazoxymethanol-β-d-glucoside (cycasin, the principal toxin), a developmental neurotoxin, mutagen, and carcinogen, and the non-proteogenic amino acid β-*N*-methylamino-l-alanine (l-BMAA, a minor component present in free and bound form). Both methylazoxymethanol (MAM) and l-BMAA are metabolized to formaldehyde, a human carcinogen with neurotoxic potential (Kisby and Spencer, 2011). This review examines how MAM and formaldehyde modulate miRNA function in brain and other tissues and the relationship between the neurotoxic and carcinogenic properties of MAM at the systems-biology level. We begin with a description of the relationship of ALS-PDC with ALS, PD, and AD, and a summary of the function and known involvement of miRNAs in development and neurodegenerative diseases, and how they overlap with cancer.

### Neurodegeneration and cancer

Neurodegenerative disorders, like cancer, are progressive fatal diseases that surface clinically long after disease initiation and cellular pathology have progressed. While cancers develop from cycling cells that undergo uncontrolled cell division and migration, neurodegenerative diseases arise from the dysfunction and loss of non-cycling cells, specifically post-mitotic neurons in the brain and spinal cord. Many such neurodegenerative disorders are associated with deposition of modified, abnormally folded proteins in the neuronal cytoplasm or extracellular spaces between brain cells. The intracellular protein accumulations (“bodies”) were described by early histologists and frequently bear their names (Bunina, Lewy, etc.), while the amyloid precursor protein is cleaved by a γ secretase to form amyloid, which accumulates in extracellular plaques.

The degeneration of motor neurons in brain and spinal cord results in the clinical picture of ALS (motor neuron disease), which is characterized by progressive limb and bulbar weakness, muscle denervation, and atrophy. Spinal and hypoglossal motor neurons develop Bunina bodies, which are closely associated with deposition of cytoplasmic TAR DNA-binding protein 43 (TDP-43). PD is characterized by neuronal deposition of α-synuclein in Lewy bodies and degeneration of nerve cells in the substantia nigra that are responsible for the quality of voluntary movement. Those with PD develop a resting tremor, limb rigidity, shuffling gait, and masked facies, often accompanied by cognitive changes. Progressive loss of cognitive function is the hallmark of AD, which is featured by the intracellular accumulation of tau protein in neurofibrillary tangles and the extracellular deposition of β-amyloid protein clusters in neuritic and senile plaques, the loss of hippocampal neurons, and brain atrophy. Genetic ablation of *Dicer* in adult forebrain neurons results in abnormal tau hyperphosphorylation and neurodegeneration (Hébert et al., 2010).

Western Pacific ALS-PDC is a polyproteinopathy (dominated by pathological tau isoforms; McGeer and Steele, 2011), the various clinical forms of this disease differentially expressing most of the neuropathological features of ALS, PD, and AD. When the disease was discovered on Guam in the 1940s, the dominant clinical form was ALS among relatively young people but, as rates declined, atypical parkinsonism among the middle-aged became prominent, only later to be displaced by an AD-like dementia in the elderly. Neuropathological examination demonstrated a single nosologic entity, with pathological emphasis on the areas corresponding to the nature of the clinical disease. Overall, the changing expression, increasing age of onset, and overall decline in ALS-PDC prevalence over the past 70 years, is consistent with a disappearing environmental agent as the principal cause. A similar longitudinal trend of neurodegenerative disease has occurred for ALS-PDC among Japanese living in the Kii peninsula of Honshu Island and among the Auyu and Jaqai linguistic groups of West Papua, Indonesia. The common feature among these three genetically distinct populations is the traditional use of cycad seed for medicine and, on Guam, for food and medicine. Rates of neurodegenerative disease have declined in Guam, Japan, and Indonesia in association with the slow disappearance of these traditional practices. Environmental factors therefore appear to dominate the etiology of western Pacific ALS-PDC, and there is no compelling evidence for the participation of either inherited factors or genetic predisposition in the three affected populations. The foregoing has been reviewed recently (Kisby and Spencer, 2011).

Unknown environmental factors are thought to have a significant etiologic role in a majority of cases of ALS, PD, and AD but, in addition, there are also hereditable genetic abnormalities in a small percentage of cases in each of these neurodegenerative diseases. In some ALS cases, the mutant human gene has been inserted into mice, which subsequently express the clinical and pathological features of the human disease, including the cytoplasmic deposition of TDP-43. This protein promotes the production of a subset of precursor miRNAs through interaction with the Drosha microprocessor complex and by binding directly with the relevant primary miRNAs. Cytoplasmic TDP-43 interacts with the Dicer complex and promotes the processing of some of the precursor miRNAs by binding to their terminal loops. Since neuronal outgrowth requires the involvement of TDP-43 in miRNA biogenesis, aberrant forms of TDP-43 that precipitate in the cytoplasm may trigger events leading to the motor neuron degeneration that characterizes ALS (Kawahara, 2010; Kawahara and Mieda-Sato, 2012). While the continuous presence of the mutant gene explains how cellular dysfunction advances to trigger a progressive and eventually fatal disease, whether and how an environmental toxin might have a comparable action in ALS-PDC is an open question that has been examined (Kisby and Spencer, 2011).

The association of ALS-PDC with cycad exposure, and in particular with the genotoxin, carcinogen, and neurotoxin MAM, has for decades led to the suspicion that cancer and neurodegenerative diseases may be related disorders (Whiting, 1988), mechanistic evidence for which was presented recently and is discussed later in this paper (Kisby and Spencer, 2011; Kisby et al., 2011a). Other evidence for overlapping molecular mechanisms in cancer and neurodegenerative diseases has been summarized by Caricasole et al. (2005), Staropoli (2008), and de Strooper (2010). For example, overactivation of the Wnt-β-catenin signaling pathway is seen in several types of human malignancy, while comparable changes in this signaling pathway in the brain are linked to neurodegeneration (Caricasole et al., 2005). In the case of the gene (*ATM*) for the inherited autosomal recessive disorder ataxia telangiectasia, DNA-damage-related loss-of-function mutations trigger both cerebellar neuronal degeneration and increase the susceptibility to neoangiogenesis and tumor progression in breast cancer (Gatti et al., 1988; Cuatrecasas et al., 2006). A second example is Down syndrome, in which there is a high incidence of leukemia or lymphoma as well as the development of AD-like neuropathology in early adulthood (Levine et al., 2009). A third is provided by the gene *PARKIN*, where loss-of-function mutations lead to early-onset PD (Kumar et al., 2011). *PARK-2* is also a candidate tumor-suppressor gene that is downregulated in several tumor types, including uterine carcinoma (Veeriah et al., 2010; Mehdi et al., 2011). Others have noted a significant association between melanoma and both ALS and PD mortality (Baade et al., 2007).

MicroRNAs have been implicated in the initiation and progression of both malignant tumors and neurodegenerative disorders by either regulating common pathways associated with both diseases or by targeting genes specific to each disease (Cooper et al., 2009; Du and Pertsemlidis, 2011). The best example is the so-called Alzheimer precursor protein (APP). While an increased expression of APP is clearly established in AD (Podlisny et al., 1987; Rovelet-Lecrux et al., 2006; Theuns et al., 2006; LaFerla et al., 2007), APP is also overexpressed in numerous cancers, including colorectal cancer (Hansel et al., 2003; Ko et al., 2007; Arvidsson et al., 2008; Krause et al., 2008). In particular, miR-17-5p (discussed later), mIR-20a, and miR-106b reduce endogenous APP expression *in vitro* (Hébert et al., 2009). A single miRNA may also be involved in neurodegeneration and cancer by regulating separate pathways (Du and Pertsemlidis, 2011). For example, miR-133b is downregulated in esophageal, lung and colon cancers (Bandrés et al., 2006; Crawford et al., 2009; Hu et al., 2010; Kano et al., 2010) and, through different pathways, may play a role in PD (Kim et al., 2007). Similarly, by acting via different pathways, miR-124a has been implicated in both tumorigenesis and PD (Lujambio and Esteller, 2007; Simunovic et al., 2009). Members of the miR-29 family of miRNAs target multiple proteins involved in both neurodegenerative diseases and cancers by regulating common pathways and those specific for each condition (Du and Pertsemlidis, 2011). Downregulation of the miR-9 gene changes the stoichiometry of axonal neurofilaments (upregulates a gene coding for a heavy neurofilament subunit) in a mouse model of human spinal muscular atrophy characterized by anterior horn sclerosis, aberrant end plate architecture, and myofiber atrophy with signs of denervation (Haramati et al., 2010), while it is overexpressed in several cancer forms, including brain tumors, hepatocellular carcinomas (HCC), breast cancer, and Hodgkin lymphoma. Inhibition of miR-9 leads to derepression of *Dicer* (Leucci et al., 2012); it suppresses matrix metalloproteinase (MMP)-14 expression via binding to a site in the 3′-UTR, thus inhibiting the invasion, metastasis, and angiogenesis of neuroblastoma (Zhang et al., 2012a). miR-132 is implicated in prostate and pancreatic tumorigenesis (Park et al., 2011; Zhang et al., 2011; Formosa et al., 2012) and downregulated in progressive supranuclear palsy, a neurodegenerative tauopathy related to the atypical parkinsonism of ALS-PDC (Smith et al., 2011). Down-regulation of the tumor suppressors miR-34b and miR-34c has been described in PD and linked to decreased expression of parkin protein (Minones-Moyano et al., 2011). MiR-7, which negatively controls the function of α-synuclein mRNA (Mouradian, 2012), also serves as an anti-metastatic miRNA in gastric cancer by targeting Insulin-like Growth Factor-1 (IGF-1) (Zhao et al., 2012). In sum, therefore, individual miRNAs may be differentially expressed in both cancers and neurodegenerative disorders; the following addresses the possible relationship between miRNAs, MAM, cancer, and ALS-PDC.

## MAM, DNA Damage/Repair, and Link with ALS-PDC

The discovery in the 1960s of cycasin and its genotoxic aglycone MAM arose from studies of ALS among Guam Chamorros who were found to use the neurotoxic seed of cycad plants (*Cycas micronesica*) for food and topical medicine (Whiting, 1988). Medicinal use of cycad seed was later discovered in the two other ALS-PDC hotspots, namely Kii Pensinsula of Japan and the Auyu and Jaqai linguistic groups of West Papua, Indonesia (Spencer et al., 1987a,b, 2005). Epidemiological studies on Guam showed an association between neurodegenerative disease and prior use of washed cycad seed to prepare flour for food (Borenstein et al., 2007). In particular, the residual content of cycasin in cycad flour was very strongly correlated with the risk for both ALS and P-D among Chamorros (Kisby et al., 1992a; Román et al., 1995; Zhang et al., 1996).

Methylazoxymethanol is a potent alkylating agent that spontaneously breaks down into reactive molecules, notably methyldiazonium ions and carbon-centered free radicals that methylate nucleic acids at the *O*^6^-, N^7^-, and C^8^ positions of guanine (Matsumoto and Higa, 1966; Shank and Magee, 1967; Nagata and Matsumoto, 1969; Nagasawa et al., 1972; Kisby et al., 1995). MAM-induced DNA damage accounts for the long-recognized dual carcinogenic and neurotoxic properties of cycasin/MAM. Below, we show that DNA damage in the form of *O*^6^-methylguanosine is specifically associated with the ability of cycasin/MAM to disrupt murine neurodevelopment (Kisby et al., 1999, 2009) and that failure to repair this type of DNA lesion by *O*^6^-methylguanine methyltransferase (MGMT) activates cellular pathways in the rodent brain that are associated with both cancer and human neurodegenerative disease (Kisby et al., 2011a). While various types of DNA lesions, especially those associated with oxidative damage, are linked with neurodegenerative diseases (ALS, PD, AD, and ATM; Martin, 2008), the association with *O*^6-^mG lesions has not been described by others. Emerging evidence indicates that DNA damage results in the differential activation of miRNAs (Wan et al., 2011; Han et al., 2012). It appears that varying amounts of DNA damage lead to the activation of unique as well as common sets of miRNAs, suggesting that the nature and intensity of DNA damage are key factors. Although several DNA damage-responsive targets have been identified, many remain to be discovered, including those activated in the brain by environmental agents that induce DNA damage.

### Systems biology of MAM in cancer

In addition to its neurotoxic properties and its possible etiologic role in ALS-PDC, MAM is an established hepatotoxin and experimental carcinogen (Adamson, 1989). Rodents that have been chronically treated with the MAM precursor azoxymethane (AOM) are widely used as models for investigating the pathogenesis and chemoprevention of human colon carcinoma (Rosenberg et al., 2009). A remarkable number of miRNAs exhibits differential expression in colon cancer tissues; these miRNAs alter cell proliferation, apoptosis, and metastasis through their interactions with intracellular signaling networks (Schetter and Harris, 2011; Wu et al., 2011). For example let-7, miR-18a and miR-143 are strongly linked to KRAS knockdown and activation of the epidermal growth factor receptor-mitogen activated protein kinase (EGFR-MAPK) pathway, whereas miR-21 and miR-126 are associated with augmentation or inactivation of the phosphatidylinositol-3-kinase pathway (Aslam et al., 2012). Activation of these downstream pathways results in autonomous tumor cell growth, increased cell survival, and initiation of angiogenesis.

The molecular mechanisms underlying the formation of colon tumors in the AOM rodent model of intestinal adenocarcinoma are solely triggered by MAM (the cytochrome P4502E1-mediated metabolite of AOM; Nigro, 1985; Chen and Huang, 2009). In this rodent model, the expression of 27 miRNAs is significantly (>6-fold) increased (e.g., miR-1, miR-34a, 132, 223, and 224), while that of 19 miRNAs is reduced (<0.49-fold; e.g., miR-192, 194, 215, and 375) in the colon tumors (Davidson et al., 2009). Epidermal growth factor receptors (EGFR) suppress the tumor suppressors miR-143 and miR-145, which coordinately control multiple targets of downstream cell-signaling pathways (i.e., K-Ras or MYC, cdk6, E2F3, and G1/S-specific cyclin-D2 or CCND2) in the AOM rodent model (Zhu et al., 2011). These studies indicate that AOM induces global changes in the expression of miRNAs in colonic tumors. MAM-induced mutation of *KRAS* (i.e., transversion from G:C to A:T at codon 12 derived from *O*^6^-mG lesions) activates this pathway and the downstream MAPK and phosphoinositide 3-kinase/Akt (PI3K/Akt) mediators, indicating that MAM perturbs gene expression in these pathways by a DNA damage-dependent mechanism. The activation of these same pathways by associated miRNAs in human colon cancer and the AOM animal model suggests that the DNA damage produced by MAM might perturb cell-signaling pathways by altering the biogenesis of miRNAs. In support, miR-1 is altered in the colon of AOM-treated rats (Davidson et al., 2009) and was a prominent hub among the 362 genes that were differentially expressed in the brain of *Mgmt*^−/−^ mice after systemic administration of MAM (Kisby et al., 2011a).

### Systems biology of MAM in brain

#### Developmental neurotoxicity of MAM

MicroRNAs play an important role in normal development of the brain, where they dictate neuronal cell identity, neurite growth, synaptic development, and neuronal plasticity (Wheeler et al., 2006; Feng and Feng, 2011; Olde Loohuis et al., 2012), and in neurodevelopmental disorders (Singh, 2007; Chang et al., 2009). Wnt1-Cre-mediated loss of Dicer, the key enzyme for miRNA biosynthesis, results in reduced expression of β-catenin together with malformation of the midbrain and cerebellum, and failure of neural crest and dopaminergic differentiation (Liu et al., 2011). Deficiency of Dicer is associated with progressive loss of miRNAs, followed by cerebellar degeneration and development of ataxia (Schaefer et al., 2007). Dicer also appears to be a target of several environmental mutagens resulting in the interference of miRNA maturation (Ligorio et al., 2011). The latter studies suggest that environmental agents that damage DNA might indirectly alter brain development by perturbing the maturation of miRNAs.

Although the involvement of miRNAs has yet to be defined, the cerebellum is reproducibly perturbed when MAM or its glucoside cycasin is administered to rodents. MAM kills or arrests neuroblasts undergoing mitosis. Rodents treated with MAM acetate *in utero* or within days of birth show strikingly abnormal development of the cerebellum associated with partial destruction of the external granular layer (Hirono and Shibuya, 1967; Shimada and Langman, 1970; Jones and Gardner, 1976; Lovell and Jones, 1980). Apoptotic cells in the external granular cell layer appear 24 h after MAM treatment, peak at 48 h and decrease at 72 h (Gobe, 1994; Lafarga et al., 1997). Pregnant rats fed crude meal containing 3% cycasin have produced litters free of brain malformations but with an increased propensity for glial malignancy in adult life (Lacqueur and Spatz, 1973). Rats exposed *in utero* [gestational day 15 (GD15) or less] to MAM or MAM acetate show microcephaly, and some develop endocrine adenomas, oligodendromas, and schwannomas, or tumors at peripheral sites (Lacqueur and Spatz, 1973). Exposure at later stages of embryonic brain development (i.e., GD17) produces an animal model that consistently displays neurochemical and neuropathological changes and associated behavioral characteristics similar to those observed in patients with schizophrenia (Featherstone et al., 2007; Lodge and Grace, 2009; Chin et al., 2011; Hradetzky et al., 2012). Calcium-signaling, glutamate receptor signaling, and long-term potentiation were the predominant cell-signaling pathways perturbed in the hippocampus of rats treated on GD17 with MAM (Lodge and Grace, 2009; Hradetzky et al., 2012). Glutamate receptor signaling and RNA post-transcriptional modification, molecular transport and RNA trafficking, were also among the top molecular networks perturbed in the prefrontal cortex of rats exposed on GD17 to MAM (Merker et al., 2009).

**Figure 1:**
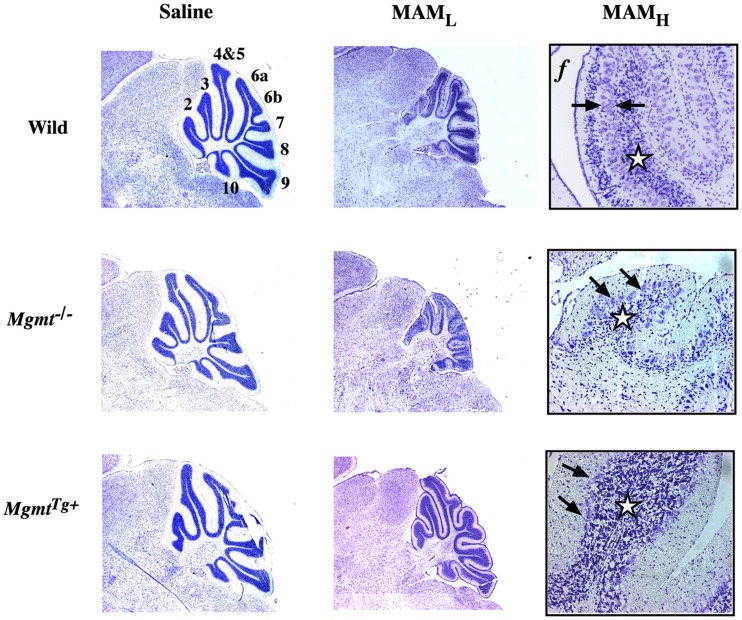
**Cytoarchitecture of the cerebellum of wild-type and DNA-repair mutant mice treated with MAM acetate**. Light micrographs of representative areas from cresyl violet-stained parasagittal sections (10 μm) of the postnatal 22-day-old cerebellum from C57BL/6J (wild), *Mgmt*^−/−^, or *Mgmt^Tg+^* treated on postnatal day 3 with a single injection of saline (*left panels*) or MAM 325 μmol, s.c. (*center panels*). Higher magnification of the cerebellum from wild-type or DNA repair mutant mice treated with MAM (*right panels*). MAM_L_, low magnification (3.85×), MAM_H_, high magnification (77×), *f*, folia. Arrows indicate disorganization of Purkinje cell layer and stars denote reduced density of neurons in granule cell layer. *Mgmt*: gene coding for *O*^6^-mG-methyltransferase (MGMT), which is knocked out in *Mgmt*^−/−^ and overexpressed in *Mgmt^Tg+^* mice (modified from Kisby et al., 2009).

The Kisby laboratory examined the relationship between MAM-induced DNA damage (*O*^6^-methylguanosine) and the development of the brain of wild-type neonatal mice and mutant mice that either lacked the *O*^6^-mG DNA-repair enzyme (*Mgmt*^−/−^) or contained genes coding for both human and murine MGMT and thus overexpressed the DNA-repair enzyme (*Mgmt*^Tg+^; Figure 1). In wild-type mice, MAM reduced the density of neurons in the granule cell layer and disrupted the organization of the Purkinje cell layer. Changes in granule cell and folia development were significantly greater in *Mgmt*^−/−^ mice treated with MAM. By contrast, cerebellar morphology was better preserved in *MGMT*^Tg+^ mice treated with MAM. Thus, MGMT protects immature neurons from MAM-induced injury through a DNA-damage-mediated mechanism (Kisby et al., 2009).

There were 251 genes differentially expressed in the cerebellum of neonatal MAM-treated *Mgmt*^−/−^ mice when compared with the corresponding cerebellum of MAM-treated *Mgmt^Tg+^* animals (G. E. Kisby, unpublished data). Glutamate and γ-aminobutyric acid neurotransmission, chromatin remodeling, and neuronal and glial development, were the predominant cellular systems perturbed by MAM in the cerebellum of *Mgmt*^−/−^ mice; together, they explain the MAM-induced changes in cerebellar development and motor function (Kisby et al., 2009). The pronounced effect of MAM on both fetal and neonatal glutamatergic neurotransmission and neural development, which are altered in schizophrenia (Kantrowitz and Javitt, 2012), suggests the developing brain is especially sensitive to DNA damage. Since the processing of mRNA and the chromatin remodeling machinery were also key targets of MAM in the fetal and neonatal brain, these studies suggest that MAM alters the development of neurons by a mechanism involving both DNA damage and epigenetic mechanisms.

#### Adult brain response to MAM

The adult human brain has a low or absent capacity to repair alkylation-induced DNA damage (Silber et al., 1996; Bobola et al., 2007), with implications for long-term survival and eventual degeneration of nerve cells (Robison and Bradley, 1984). We tested the hypothesis that the DNA-damaging properties of MAM, which are mutagenic and tumorigenic in cycling cells of the colon epithelium (Rosenberg et al., 2009), activate molecular networks associated with the degeneration of post-mitotic neurons in human neurodegenerative disease. To address this hypothesis, we compared the relationship between MAM-induced DNA damage (*O*^6^-methylguanosine) and gene expression patterns in the brains of adult wild-type and *Mgmt*^−/−^ mice. Two separate laboratories treated groups of mice with a single systemic dose of MAM, and the combined data were mined for common brain transcriptional profiles. A third laboratory conducted blinded analyses of brain *O*^6^-methylguanosine levels. Signaling pathways associated with cancer and human neurodegenerative disease were activated in the mature mouse brain as the result of unrepaired MAM-induced DNA damage (Kisby et al., 2011a).

A computational approach was used to determine which miRNAs are likely to regulate MAM-modulated gene expression in the adult mouse brain. The most significant molecular networks derived from 362 MAM-triggered, differentially expressed genes revealed hubs involving NF-κB (nuclear factor of kappa light polypeptide gene enhancer in B-cells), calcium-binding proteins (i.e., calcineurin, calmodulin), brain-derived neurotrophic factor (BDNF), glutamate receptors *N*-methyl-d-aspartate (NMDA), and α-amino-3-hydroxy-5-methyl-4-isoxazolepropionic acid (AMPA), cyclic AMP response element-binding factor (CREB), and miR-1 (Kisby et al., 2011a). Each of these gene hubs was computationally analyzed to identify potential regulating miRNAs. Specifically, this analysis required the use of the miRNA database (miRDB, www.mirdb.org) to predict miRNAs with seed regions that match the base pair sequences of the mRNAs within their 3′-UTRs. miRNAs that controlled more than one mRNA are listed in Table 1. These data demonstrate that multiple miRNAs likely regulate the MAM-generated hubs, a finding that has been shown by others (Nohata et al., 2012).

**Table 1:** **miRNAs predicted to regulate genes linked to MAM-induced DNA damage (*O*^6^-methylguanosine) in the brains of MAM-treated mice lacking the DNA-repair enzyme *O*^6^-methylguanine methyltransferase (based on data in Figure 3 of Kisby et al., 2011a)**.

miRNA	Gene targets
miR-134	Brain-derived neurotrophic factor (*BDNF*) and cAMP response element-binding factor (*CREB*)
miR-204	*BDNF* and α-amino-3-hydroxy-5-methyl-4-isoxazolepropionic acid (*AMPA*)
miR-211	*BDNF* and *AMPA*
miR-505	*BDNF* and *AMPA*
miR-590-3p	*BDNF* and *CREB*

MiR-134 has an important role in the brain, where it is essential for activity-dependent dendritic outgrowth, nerve growth cone guidance, and size of dendritic spines of hippocampal neurons (Schratt et al., 2006; Khudayberdiev et al., 2009; Han et al., 2011). Silencing of miR-134 expression reduces dendritic spine density and renders mice refractory to seizures and hippocampal injury caused by *status epilepticus* (Jimenez-Mateos et al., 2012). MiR-134 is also a powerful inducer of pluripotent stem cell differentiation and functions as a regulator of cell proliferation, apoptosis, and migration during lung development (Zhang et al., 2012c). The expression of miR-134 is reduced in gastrointestinal stromal tumors (Haller et al., 2010). MiR-204 is proposed to play critical roles in the development and progression of malignant peripheral nerve sheath tumors (Gong et al., 2012); it also suppresses tumor cell growth of renal clear cell tumors (Mikhaylova et al., 2012). Downregulation of miR-204 activates Ras in gastric cancer cells (Lam et al., 2011) and reduces lung metastasis of squamous cell carcinomas of the head and neck (Lee et al., 2010). Transfection of miR-204 into human trabecular meshwork cells increased levels of apoptosis, decreased viability, and increased the accumulation of oxidized proteins, decreased induction of endoplasmic reticulum stress response markers, and reduced expression of inflammatory mediators (Li et al., 2011). MiR-211 has a binding site for angiopoietin-1, which is a vascular strengthening factor during vascular development and a protective factor for pathological vascular inflammation and leakage, including brain vascular leakage as occurs in stroke (Chen et al., 2010). MiR-211 expression promotes colorectal cancer growth by down-regulating expression of the CHD5 tumor suppressor (Cai et al., 2010). Increased miR-211 expression is linked with progression and vascular invasion of oral carcinoma (Chang et al., 2008). MiR-211 is also implicated as a tumor suppressor for melanoma invasion (Levy et al., 2010). MiR-505 acts as a tumor suppressor by inhibiting proliferation and inducing apoptosis of human breast cancer cells (Yamamoto et al., 2011). MiR-590-3P regulates the transcription of heterogenous nuclear ribonucleoprotein A1 (hnRNP-A1); both are dysregulated in the blood of patients with AD but not with frontotemporal lobal degeneration (Villa et al., 2011).

The differential response of genotypes to MAM vs. vehicle were grouped together with the response of *Mgmt^−/−^* brains to systemic treatment with MAM vs. vehicle, for a total of 443 non-duplicated genes. The most significant scoring sub-network of these MAM-differentially expressed genes (*p* < 10^-46^) contained hubs for F-actin, NF-κB, cofilin, calcium/calmodulin-dependent protein kinase II (CaMKII), glycogen synthase, the AMPA receptor, BDNF, and miR-1. There is a large literature on miR-1, much of which is focused on cardiac muscle function (Mishima et al., 2007). MiR-1 is present in nerve cells, at least in the peripheral nervous system (Bastian et al., 2011), and blood miR-1 expression has been used to distinguish normal subjects from patients with PD (Margis et al., 2011). MiR-1 is also involved in late-stage cartilage cell differentiation (Sumiyoshi et al., 2010). In prostate gland cells, miR-1 is a candidate tumor suppressor and is frequently downregulated in various types of cancer (Hudson et al., 2012). Available evidence suggests that miR-1 alters the cellular organization of F-actin, thereby inhibiting filopodia formation, cell motility, and tumor invasion. MiR-1 also has an oncosuppressive role in breast, lung, thyroid, liver, renal, and colorectal cancer (Datta et al., 2008; Beltran et al., 2011; Leone et al., 2011; Kawakami et al., 2012; Kojima et al., 2012; Migliore et al., 2012), and, in the latter, this activity is silenced by miR-1 methylation (Suzuki et al., 2011).

Ingenuity^®^ pathway analysis (IPA) was used to identify the most significantly enriched biofunctions for each data set by combining significant MAM-associated brain gene expression changes at all time-points and comparing these data. IPA analysis of the 443 gene set revealed (a) Nervous System Development and Function (64 genes), Embryonic Development (22 genes), and Organ Development (14 genes) as the top three IPA Physiological Systems Development and Functions perturbed by MAM, while (b) Neurological Disorders (159 genes), Psychological Disorders (75 genes), Cancer (114 genes), and Genetic Disorders (212 genes) were the four most significant diseases and disorders altered in the brains of MAM-treated vs. vehicle-treated *Mgmt^−/−^* mice plus *Mgmt^−/−^* vs. wild-type mice treated with respect to MAM vs. vehicle (Kisby et al., 2011a).

The Kyoto Encyclopedia of Genes and Genomes (KEGG) was used to determine the top KEGG pathways perturbed by MAM in either wild-type or *Mgmt^-^/-*brains. Pathways involved in cancer (13 genes), Wnt signaling (10 genes), insulin-signaling pathway (9 genes), purine signaling (8 genes), and MAPK signaling (7 genes) were among the most significant. Other prominent KEGG pathways included those involving neurotrophin signaling (6 genes), focal adhesion (6 genes), chemokine signaling (5 genes), neuroligand-receptor interaction (5 genes), and the calcium-signaling pathway (4 genes). Most of these pathways have been implicated in AD and/or colon cancer (Kisby et al., 2011a) and, in a separate recent study, some (pathways in cancer, Wnt signaling, MAPK signaling, and calcium-pathway signaling) have been predicted to be regulated by miR-1/miR-133A (Table 2).

**Table 2:** **Top KEGG pathways in the brains of MAM-treated mice and associated miRNAs in human cancers**.

Top MAM-associated KEGG pathways in mouse brain	Genes	Phenotype	miR-1/miR- 133A-regulated in human cancers*
Pathways in cancer	13	CC	Yes
Wnt signaling	10	AD, CC	Yes
Insulin signaling	9	AD, ALS	
Purine metabolism	9		
Prostate cancer	8	CC	
MAPK signaling	7	AD, CC	Yes
Melanogenesis	6	PD? CC	
Neurotrophin signaling	6	AD	
Focal adhesion	6	AD, CC	Yes
Chemokine signaling	5	AD	Yes
Neuroactive ligand-receptor interaction	5	AD	
Calcium-signaling pathway	4	AD, CC	Yes

Top brain KEGG pathways (derived from 443 MAM-modulated genes) and the number of MAM-modulated genes and human diseases associated with each pathway. AD, Alzheimer disease; ALS, amyotrophic lateral sclerosis; CC, colon cancer; PD, idiopathic Parkinson’s disease (modified from Kisby et al., 2011a). The right-hand column shows the biological processes or signaling pathways potentially regulated by the miR-1/miR-133a cluster in human cancers examined by *Nohata et al. (2012). Additional signaling pathways in Nohata and colleagues’ top 20 included those related to the glutamatergic synapse and axon guidance, both of which are perturbed by MAM.

## Metabolism of MAM and L-BMAA to Formaldehyde

**Figure 2:**
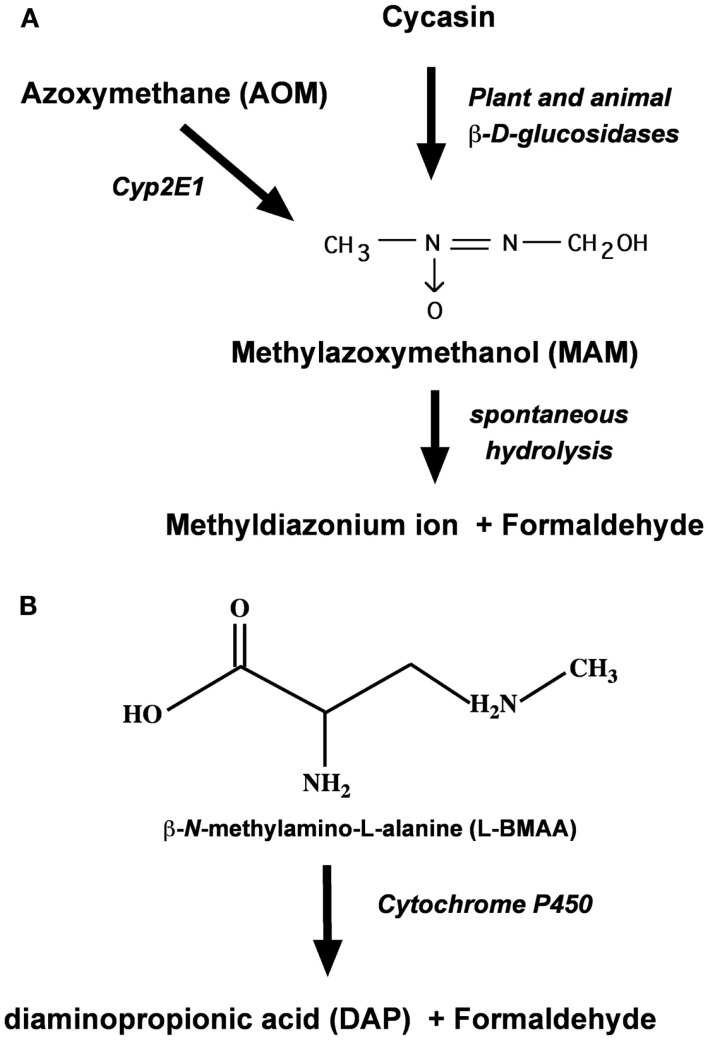
**Metabolism of the cycad toxins cycasin, MAM, and l-BMAA by plant and animal tissues**. **(A)** Enzymatic conversion of cycasin or azoxymethane to MAM and its degradation product formaldehyde. **(B)** Enzymatic *N*-demethylation of l-BMAA by brain P450 to formaldehyde.

Cycasin is but one of several cycad azoxyglycosides, all of which release MAM upon enzymatic hydrolysis (Kisby et al., 1999; Figure 2). Cycasin and the other azoxyglycosides are converted to MAM by plant and animal β-glucosidases, whereas AOM is converted to MAM by mixed function oxidases (i.e., P450 Cyp2E1). At physiological pH, MAM is unstable and spontaneously breaks down to formaldehyde and the methyldiazonium ion, the proximate metabolite that is responsible for the facile methylation of cellular macromolecules, including nucleic acids. Alternatively, MAM may also undergo oxidation by Cyp2E1 or alcohol dehydrogenase in the liver or extrahepatic organs (e.g., brain) to form methylazoxyformaldehyde, which under physiological conditions spontaneously degrades to release formic acid and methyldiazonium ion (Sohn et al., 2001). Since DNA alkylation was significantly greater in the colon, kidney, and lung of MAM-treated Cyp2E1-null than wild-type mice, but reduced after similar treatment with AOM (Sohn et al., 2001), the enzymatic oxidation of MAM is not likely to be the predominant metabolic pathway in both hepatic and extrahepatic organs.

Whereas MAM-glycone (cycasin) is the principal toxic component of cycad seed (4% w/w) that can damage the nervous system, a second agent, l-BMAA, is also significant because this excitant amino acid induces a motorsystem disease in orally fed primates (Spencer et al., 1987c). Present in cycad seed in concentrations 10-fold lower than that of cycasin (Kisby et al., 1992a), l-BMAA is also found in terrestrial, freshwater, and marine environments (Banack et al., 2007; Purdie et al., 2009; Brand et al., 2010; Mondo et al., 2012). The rapid uptake, accumulation, and release of l-BMAA in cortical explants and synaptosomes (Kisby and Spencer, 2011) suggest that the sub-chronic neurotoxic effects of this cycad toxin are related to unknown intracellular actions. Like MAM and AOM, l-BMAA is metabolized to toxic species in rat tissue slices, mouse cortical explants, and rat crude cerebral microsomes (Kisby et al., 1992b). Kisby et al. (1992c) showed that microsomes and mitochondria from the rodent brain metabolized both l-BMAA and aminopyrine (standard substrate for *N*-demethylation by cytochrome P450) to formaldehyde (Figure 2), enzymatic reactions that were inhibited by the aminopyrine *N*-demethylase inhibitors deprenyl, SKF525A, and piperonyl butoxide (Kisby et al., 2011a).

In sum, therefore, cycad-associated l-BMAA and MAM are both metabolized to formaldehyde; however, the role of formaldehyde in MAM- or l-BMAA-induced neurotoxicity, or in the tumorigenic activity of MAM, is not known.

### Formaldehyde, cancer, neurotoxicity, and neurodegenerative disease

Formaldehyde is a skin, eye, airway, and throat irritant, and a cause of allergic contact dermatitis. Epidemiological studies have shown that formaldehyde exposure increases the risk of childhood and adult asthma (Wieslander et al., 1997; Rumchev et al., 2002), acute respiratory tract illness (Tuthill, 1984), nasopharyngeal cancer (Vaughan et al., 2000) and, possibly, leukemia (Zhang et al., 2010a). There is also a statistically non-significant increase in brain tumors with “ever embalming” among formaldehyde-exposed embalmers and funeral directors. In animal studies, strong links have been made between formaldehyde exposure and nasal carcinoma (Kerns et al., 1983). Furthermore, the International Agency for Research on Cancer (IARC, 2006) has classified formaldehyde as a human carcinogen. One of us (Glen E. Kisby) recently reported that genotoxins in cigarette smoke, in which formaldehyde is predominant, induce various types of DNA damage (single- and double-strand breaks; DNA-protein crosslinks) in the brains of mice, with coincident changes in the ratio of tau isoforms and phosphorylated tau (La Maestra et al., 2011). Formaldehyde can alkylate DNA to form adducts, inhibit the expression of MGMT (which specifically repairs *O*^6^-mG DNA lesions; Grafstrom et al., 1985), crosslink DNA and protein, and bind to albumin or the *N*-terminal valine of hemoglobin. In cycling cells, failure to repair *O*^6^-mG lesions leads to mutations (G:C to A:T) and the activation of cellular pathways that culminate in tumorigenesis.

Formaldehyde also has poorly understood acute and chronic neurotoxic effects. Insomnia, memory loss, mood alterations, nausea, and fatigue have been reported with low airborne formaldehyde concentrations. Prolonged occupational exposure to formaldehyde is associated with somnolence, headache, seizures, mood instability, memory, and cognitive deficits. Effects in controlled animal studies include neurobehavioral changes and neurotoxicity (learning and memory deficits). Levels of superoxide dismustase-1 (which is mutated in some cases of dominantly inherited ALS) and glutathione peroxidase decreased significantly in the prefrontal cortex of rats treated parenterally with formaldehyde (National Toxicology Program, 2010). Other information links formaldehyde with neurodegenerative diseases related to ALS-PDC, but definitive evidence is lacking, as summarized below.

#### Amyotrophic lateral sclerosis

Exposure to cigarette smoke, of which formaldehyde is the major contaminant, has been strongly linked to ALS risk in two large epidemiological studies (Weisskopf et al., 2005; Gallo et al., 2009). Both found significant trends with smoking duration, with prolonged and current smoking increasing ALS risk by two- to threefold. Time since smoking cessation was associated with a decreased risk for ALS relative to those who continued smoking. Gallo et al. (2009) pinpointed formaldehyde as the most likely culprit in cigarette smoke, but direct evidence is lacking. Formaldehyde was also singled out for further research in a large prospective study of chemical exposures and ALS, which found strongly significant dose-response relationships with increasing years of smoking exposure (Weisskopf et al., 2005, 2010).

#### Alzheimer disease

Chinese investigators have explored the status of formaldehyde in AD; they report that formaldehyde levels are increased in 20–40% of patients with senile dementia (He et al., 2010). These investigators found that human endogenous levels of formaldehyde are maintained at a low concentration (0.01–008 mmol/l blood) under physiological conditions, with concentrations increasing during aging (>65 years). Importantly, urine formaldehyde concentrations of normal elderly volunteers are significantly lower than those of elderly patients with AD, and the elevated urinary levels of formaldehyde are inversely correlated with Mini Mental State Examination (MMSE) scores and hippocampal formaldehyde concentrations in AD patients (He et al., 2010; Tong et al., 2011). A correlation between memory loss and brain tissue formaldehyde levels was also observed in wild-type mice treated with formaldehyde (He et al., 2010; Tong et al., 2011). Taken in concert, these data suggest that formaldehyde may have a role in AD.

#### Parkinsonism

Chemicals that induce parkinsonism in humans and/or animals include: methanol, which is oxidized to formaldehyde; methylphenyltetrahydropyridine, which is formed from formaldehyde, methylamine, and α-methylstyrene; rotenone, which inhibits mitochondrial formaldehyde oxidation, and cycad flour, which contain formaldehyde-forming neurotoxins (Shen et al., 2010). That methanol intoxication can precipitate parkinsonism is long recognized, and a dozen reports describe cases of l-DOPA-sensitive and -refractory methanol-induced extrapyramidal disease (and one case of multiple system atrophy) over the past 40 years. Methanol is produced endogenously by hydrolysis of the methylester bonds of proteins that are methylated by the endogenous methyl donor *S*-adenosylmethione (SAM). SAM increases formation of methanol, formaldehyde, and formic acid in striatal homogenates, and formaldehyde is more toxic to neurons than glial cells. Farnesyl-l-cysteine analogs, which inhibit SAM methylation, block parkinsonian signs (tremor, rigidity, abnormal posture, and hypokinesia) when SAM is injected into the rat brain. Lee et al. (2008) proposed that a SAM-mediated increase in formaldehyde has a possible role in PD-like neuronal damage and the aging process.

### Systems biology of formaldehyde in non-neural cells

The Fry laboratory has recently investigated whether formaldehyde exposure disrupts miRNA expression levels within lung cells (Rager et al., 2011). Human A549 lung epithelial cells were exposed to formaldehyde (1 ppm) using an *in vitro* exposure system that physically replicates *in vivo* human lung gas exposures (Bakand et al., 2005). Upon exposure to formaldehyde, the lung epithelial cells showed decreased expression in 89 of 534 miRNAs that were measured using human miRNA microarrays (version 1). All of the modulated miRNAs were downregulated by formaldehyde exposure. This general trend of miRNA down-regulation has been observed in rat lung cells exposed to cigarette smoke (Izzotti et al., 2009), as well as in multiple tumor cell types, including lung cancer, breast cancer, and leukemia (Lu et al., 2005).

A detailed analysis was made on the four most significantly down-regulated miRNAs, namely miR-33, miR-330, miR-181a, and miR-10b, as determined through microarray analysis and qRT-PCR. These miRNAs have been studied to some extent, and knowledge about their regulation and association to disease is growing. For example, the expression level of miR-33 is decreased in tissues from patients with lung carcinoma (Yanaihara et al., 2006). In addition, miR-330 expression levels are reduced in human prostate cancer cells when compared with non-tumorigenic prostate cells (Lee et al., 2009). Furthermore, miR-330 has been suggested to act as a tumor suppressor by regulating apoptosis of cancer cells (Lee et al., 2009). In addition, miR-10b shows altered expression levels within breast cancer tissue and is one of the most consistently dysregulated miRNAs able to predict tumor classification (Iorio et al., 2005; Ma et al., 2007). These findings suggest that miR-33, miR-330, and miR-10b may influence cellular disease state, specifically related to cancer. Formaldehyde exposure also altered the expression level of miR-181a, which has known associations with leukemogenesis (Marcucci et al., 2009). The specific link between formaldehyde exposure and leukemia is currently debated, as numerous epidemiological studies show evidence for possible association with this disease (Pinkerton et al., 2004; Zhang et al., 2010b), as well as against it (Marsh and Youk, 2004; Bachand et al., 2010). Nevertheless, the dysregulation of miR-181a upon exposure to formaldehyde is of interest.

A systems-biology approach was used to understand the potential biological implications of the miRNA expression changes induced in lung cells by acute formaldehyde exposure. A stringent computational matching approach was used to identify predicted mRNA targets for miR-33, miR-330, miR-181a, and miR-10b. The identified mRNA targets were used to construct molecular networks and were analyzed for their known involvement in signaling pathways and biological functions. The identified networks showed enrichment for various canonical pathways, including NF-κB and interleukin-8 (IL-8) signaling. Although very few predicted targets overlapped among the four miRNAs, proteins involved with cancer mechanisms (including that of the NF-κB pathway) were found within the miRNA target networks. Importantly, NF-κB has clear links to inflammation and cancer development (Karin and Greten, 2005; Schmid and Birbach, 2008). Also related to inflammation, IL-8-related signaling molecules were present in the miRNA target networks. Previous studies have shown IL-8 release in lungs cells representing an inflammatory response after exposure to other air pollutants (Jaspers et al., 1997; Sexton et al., 2004). In addition, investigations have shown increased IL-8 levels in lungs of patients with diseases such as acute lung injury (McClintock et al., 2008), adult respiratory distress syndrome (Jorens et al., 1992), and asthma (Bloemen et al., 2007). Inflammation is a recognized formaldehyde-induced response, as formaldehyde is known to irritate the respiratory system (Tuthill, 1984) and increase asthmatic response (Wieslander et al., 1997; Rumchev et al., 2002). The new findings suggest that the canonical pathways associated with formaldehyde-induced miRNA alterations may impact the regulation of biological pathways associated with various disease states, including cancer and inflammation (Rager et al., 2011). There is also a vast literature on the possible pathogenic role of inflammation in neurodegenerative diseases, including ALS, PD, AD, and ALS-PD, the subject of the present paper (McGeer and McGeer, 1998, 2001; Lewis et al., 2012; Tufekci et al., 2012). A neuroinflammatory response was suggested by the presence of transcriptional changes in extracellular-matrix-receptor interaction (four genes upregulated, one gene downregulated) and cytokine–cytokine receptor interaction (three genes upregulated, two genes downregulated) in the brains of adult *Mgmt*^−/−^ mice treated 6 months earlier with a single dose of MAM (Kisby et al., 2011b).

The data obtained with the lung epithelial cells were compared with those of an existing genomics database (e.g., mRNA) from a study that evaluated human lung cells exposed to formaldehyde (Li et al., 2007). Using the predicted targets in the most significant miRNA networks, the following genes showed overlap: *BDNF*, *BMPR2*, *CACNA1C*, *CSNK1D*, *HMGA2*, *HSF2*, *HSPH1*, and *PIM1*. These genes have been shown to play a role in various diseases. For example, *BDNF* modulates neurogenesis after injury to the central nervous system (Ming and Song, 2005). *CSNK1D*, or casein kinase 1δ, is upregulated in breast cancer tissue (Abba et al., 2007). *HMGA2*, or high mobility group AT-hook 2, is oncogenic in many cells, including lung carcinoma cells, and is regulated by the tumor-suppressive miRNA let-7 (Lee and Dutta, 2007). Lastly, the serine-threonine protein kinase gene *PIM1*, or Pim-1 oncogene, is found at increased levels within prostate cancer tissue (Dhanasekaran et al., 2001). Network analysis of all formaldehyde-responsive genes identified through the Li et al. (2007) study revealed significant associations with cancer, inflammation, and endocrine system regulation, which also overlap with the findings of Rager et al. (2011). These genes are therefore linked with formaldehyde-induced changes in miRNA abundance as well as mRNA alterations, and they are related to a diverse range of cellular responses including tumorigenesis.

In summary, the new data provide evidence of a potential mechanism that may underlie the cellular effects induced by formaldehyde, namely an alteration in miRNA expression. The set of 89 miRNAs was changed in human lung cells exposed to formaldehyde. Mapping the most significantly changed miRNAs to their predicted mRNA targets and their network interactomes within the cell revealed the association of formaldehyde exposure to inflammatory response pathways. Future research will investigate whether the expression levels of these miRNAs may serve as potential biomarkers of formaldehyde exposure in humans. Such biomarkers can be utilized to better monitor human exposure to environmental toxicants and their related health effects. Based on these findings, it was suggested that miRNAs likely play an important role in regulating formaldehyde-induced gene expression and may represent a possible link between exposure and disease. Whether these results can be extrapolated to tissues other than lung, notably to brain tissue, is unknown. However, since formaldehyde is also a metabolite of MAM (and l-BMAA), it is of interest to determine if the modulation of miRNAs by formaldehyde in the lung can be related to brain transcriptional changes induced by systemic exposure to MAM.

### Correlation of miRNAs in formaldehyde, MAM, cancer, and neurodegeneration

To identify miRNAs that may mediate MAM-associated gene expression, we examined all genes that were altered in expression in the most significant brain expression network published in our earlier study of transcriptional changes induced in adult wild-type mice vs. *Mgmt*^−/−^ (see Figure 3 from Kisby et al., 2011a). All genes whose expression was altered by MAM vs. vehicle in the *Mgmt*^−/−^ mice were examined for miRNA seed matches. The miRNA database (miRDB, www.mirdb.org) was used to predict miRNAs with seed regions that match the base pair sequences of the mRNAs within their 3′-UTR (Wang, 2008; Wang and El Naqa, 2008). There were 18 genes for which one or more miRNAs was identified. A comparison of all 89 formaldehyde-modulated miRNAs in human lung epithelial cells (Rager et al., 2011) with the miRNAs predicted here to regulate genes in the brains of MAM-treated *Mgmt*^−/−^ mice show overlap of 6 miRNAs: miR-107, miR-152, miR-17-5p, miR-181d, and miR-454-3p. MiR-107 promotes tumor progression and is linked to AD (Chen et al., 2011; Augustin et al., 2012). MiR-152 inhibits proliferation of ovarian cancer cell lines and is regulated in axon guidance (Liu et al., 2012; Zhou et al., 2012).

**Figure 3:**
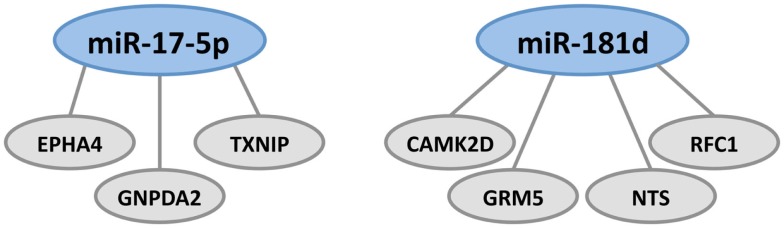
**Formaldehyde-responsive miRNAs predicted to modulate MAM-associated genes in the brains of *Mgmt*^−/−^ mice**. These include miR-17-5p and miR-181d, which regulate genes involved in tumor suppression, DNA repair, amyloid deposition, and glutamatergic and dopaminergic neurotransmission. MiR-17-5p regulates the expression of *EPHA4*, *GNPDA2*, and *TXNIP*. There is a large brain-related literature on the gene encoding ephrin type-A receptor 4 (*EPHA4*) which, in the hippocampus, is located in the neuropil layers of CA1, CA3, and dentate gyrus. *EPHA4* is involved in axonal development, maturation, targeting, and synapse formation (Tremblay et al., 2007; Clifford et al., 2011). Inhibition of EPHA4 signaling reduces apoptosis in hippocampal CA1 neurons and is involved in the γ-secretase pathway, which processes APP to the extracellular amyloid deposits that characterize AD, ALS-PDC, and other tauopathies (Inoue et al., 2009). EphA4 and EphB2 receptors were reduced in the hippocampus before the development of impaired object recognition and spatial memory in an AD mouse model of cognitive decline that overexpresses human APP protein (Simón et al., 2009). Literature on the gene coding for glucosamine-6-phosphate deaminase 2 (2-amino-2-deoxy-d-glucose 6-phosphate; *GNPDA2*) is primarily related to obesity; the gene is downregulated in the hypothalamus in rats on a high fat diet (Gutierrez-Aguilar et al., 2012). The gene encoding thioredoxin-interacting protein (*TXNIP*) is a molecular nutrient sensor important in the regulation of energy metabolism and also involved in obesity (Blouet and Schwartz, 2011). Blockade of the NMDA receptor upregulates *TXNIPin vivo* and *in vitro*, where it binds thioredoxin and promotes vulnerability to oxidative damage (Martel et al., 2009). Transcriptional expression of both *TXNIP* and *TP53* is upregulated in Cockayne syndrome, a human premature aging and dementing disorder (without amyloid deposition) associated with neurological and developmental abnormalities, and caused by mutations mainly in the CS group B gene (*ERCC6*, excision repair cross-complementing rodent repair deficiency 6; de Sousa Andrade et al., 2012). MiR-181d regulates the expression of *CAMK2D*, *GRM5*, *NTS*, and *RFC1*. The gene coding for DNA replication factor-1 (*RFC1*) is involved in DNA replication and repair, which would be expected following either MAM- or formaldehyde-induced DNA lesions. Neurotensin (NT) is a brain and gastrointestinal peptide that acts on G-coupled and transmembrane receptors that modulate dopaminergic transmission in brain pathways, notably the nigrostriatal pathway that degenerates in PD. Interactions may exist between NT receptor subtype 1 (NTS1) and dopamine D_2_ or NMDA receptors, such that NT-induced amplification of the latter may be involved in neurodegeneration (Tanganelli et al., 2012). *GRM5* codes for the metabotropic glutamate receptor 5, one of the neuronal synaptic receptors that responds to the brain’s major excitatory transmitter glutamate. This activates a G-protein-coupled response that activates a phosphatidylinositol-calcium second messenger system and generates a calcium-activated chloride current. *GRM5* is upregulated following repeated exposure to glutamate *in vitro* (Kawaai et al., 2010), downregulated in schizophrenia (Choi et al., 2009) and most probably involved in a host of degenerative disorders. MiR-181d also regulates the expression of calcium/calmodulin-dependent protein kinase II delta (*CAMK2D*).

MiR-17-5p and miR-181d, which are downregulated in formaldehyde-treated human lung epithelial cells, were predicted to regulate the greatest number of genes in the brains of MAM-treated *Mgmt*^−/−^ mice (Figure 3). MiR-181d, which regulates the three genes shown in Figure 3, down-regulates MGMT mRNA and protein expression in glioblastoma cells (Zhang et al., 2012b), is involved in MAPK signaling in pancreatic cancer cells (Ikeda et al., 2012), and is upregulated by hepatic transforming growth factor beta (TGFβ) in promoting hepatocarcinogenesis (Wang et al., 2010). MiR-17-5p targets tumor protein P53-induced nuclear protein 1 (TP53INP1), which suppresses cell growth and promotes apoptosis of cervical cancer cells (Wei et al., 2012). Behrens et al. (2009) have noted that whereas reduced P53 expression arising from mutations may lead to uncontrolled cell proliferation, as in colorectal cancer, osteosarcoma, and other tumors, increased P53 expression may activate pathways leading to cell death, such as occurs in AD. MiR-17-5p also has tumor-suppressing activity in hepatocellular, gastric, pancreatic, breast, and cervical cancer (Hossain et al., 2006; Yu et al., 2010; Chen et al., 2012; Wang et al., 2012) and is also thought to be involved in the regulation of APP expression (Hébert et al., 2009).

## Summary and Conclusion

Understanding the etiology and pathogenesis of western Pacific ALS-PDC is expected to illuminate related neurodegenerative disorders, including ALS and AD. ALS-PDC is predominantly or exclusively an environmental disorder of unknown causation but strongly associated with prior exposure to cycad seed, which contains neurotoxic compounds (cycasin/l-BMAA) that are metabolized to formaldehyde, a human carcinogen. While highly plausible, a cause-effect relationship between these agents and ALS-PDC has yet to be established.

**Figure 4:**
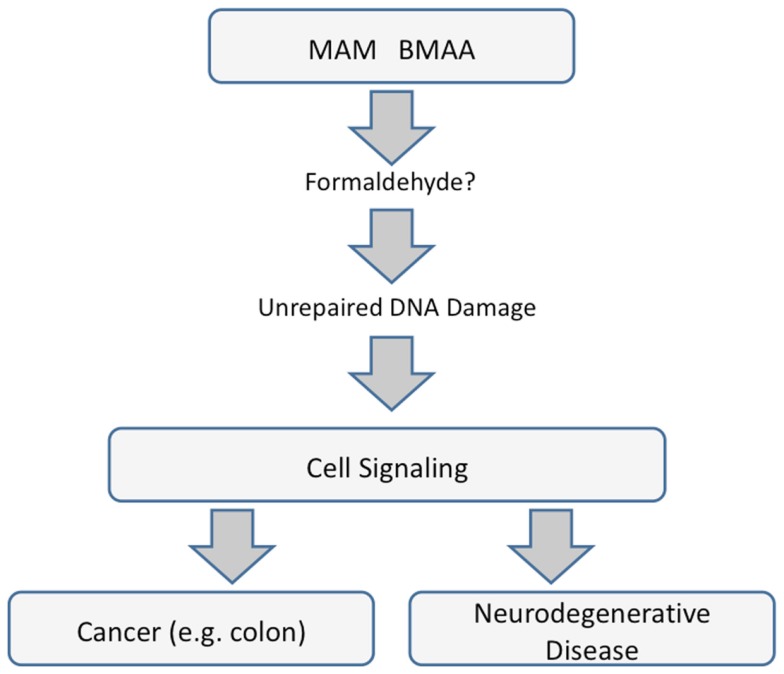
**Common pathways underlying cancer and neurodegenerative diseases that are mediated by miRNAs**.

The mechanisms underlying the neurotoxic and carcinogenic action of MAM are related and specifically tied to the modulation of signaling pathways apparently triggered by unrepaired DNA damage. Whereas MAM-induced perturbation of those pathways leads to uncontrolled division in cycling cells, in brain tissue (containing post-mitotic neurons), the primary outcome is linked to neurological diseases and perhaps to ALS-PDC, with which the MAM-glycone (cycasin) is strongly associated in epidemiological studies. Whether this is mediated by MAM directly or by formaldehyde is unknown, but clearly the mechanisms underlying MAM murine neurotoxicity and carcinogenicity are related (Figure 4).

Selected miRNAs appear to stand at the crossroads between the toxic actions of MAM and formaldehyde. Direct evidence exists to implicate a number of miRNAs in the acute actions of formaldehyde in non-neural (lung) cells. Formaldehyde-responsive miRNAs predicted to modulate MAM-associated genes in mouse brains lacking MGMT include miR-17-5p and miR-18d, which regulate genes involved in tumor suppression, DNA repair, amyloid deposition, and glutamatergic and dopaminergic neurotransmission. However, direct evidence for the involvement of specific miRNAs in MAM-induced neurotoxicity has yet to be demonstrated. This conclusion notwithstanding, there is a provocative association between top KEGG pathways in the brains of mice exposed to MAM and in human cancers, with suspicion falling heavily on a prominent role for at least one miRNA, namely miR-1. However, miRNAs are but one of at least three known mechanisms of epigenetic regulation, and there is no information on the possibility that MAM modulates brain gene expression via cytosine methylation or histone modification.

The ALS-PDC-associated cycad genotoxins MAM and cycasin are but two among a large number of chemicals, including a variety of food additives, food components, environmental contaminants, *N*-nitroso compounds, rodent carcinogens, and antineoplastic agents that have been evaluated in non-human primates for their long-term carcinogenic activity (Thorgeirsson et al., 1994). Treatment of primates with cycasin or MAM induced primarily HCC, but also renal, esophageal, and intestinal adenocarcinomas (Sieber et al., 1980; Adamson, 1989). Emerging evidence indicates that miRNA dysregulation plays a key role in HCC by promoting a number of cancer-inducing cell-signaling pathways (Law and Wong, 2011; Negrini et al., 2011). The aberrant activation of the Wnt/β-catenin signaling pathway is reportedly a significant contributor in HCC (Takigawa and Brown, 2008; Nambotin et al., 2011; Nejak-Bowen and Monga, 2011), a prominent pathway that was also perturbed by MAM in the rodent brain (Kisby et al., 2011a). Dysregulation of Wnt/β-catenin signaling is also frequently observed as an early event in the AOM-induced (MAM-mediated) rodent model of colon cancer (Takihashi and Wakabayashi, 2004; see Figure 2). Thus, HCC, colon, and other carcinomas induced by MAM in non-human primates might involve the activation of downstream cell-signaling pathways by associated miRNAs. While MAM-induced activation of the Wnt/β-catenin pathway leads to uncontrolled cell proliferation in the AOM model of colon cancer, suppression of this pathway in the brain may promote cell death. Boonen et al. (2009) propose that disrupting the tightly regulated brain Wnt signaling pathway may constitute a key pathological event in AD. They propose that amyloid-beta (Aβ), a key protein in the senile plaques of AD, may down-regulate the Wnt/β-catenin pathway, thereby upregulating GSK3β and its subsequent hyperphosphorylation of tau, linking Aβ and the neurofibrillary pathology characteristic of AD and ALS-PDC. This is consistent with recent data showing that GSK3β, GSK3α, tau oligomers, and phosphorylated and truncated forms of tau are elevated in a mouse model of AD (Kisby et al., 2011c). Others have shown that inhibition of GSKβ increases mouse brain IGF-1 (Bolós et al., 2010), which in turn promotes Aβ production (Araki et al., 2009; De La Monte, 2009). Transcriptome analysis of enzymes involved in tau phosphorylation point to the involvement of extracellular signal-regulated kinase 1 (ERK1), the gene that was perturbed in a DNA-damage-anchored manner in the brains of *Mgmt*^−/−^ mice both days and 6 months after a single systemic dose of MAM; ERK1 protein was significantly increased at the later timepoint, as was fodrin cleavage, which indicates the activation of caspase-3 (Kisby et al., 2011b), an enzyme with an important role in cleaving tau (Rohn et al., 2002). Tumor-suppressor miR-16 (downregulated in some cancers) and miR-132 (which is methylation-silenced in prostate cancer) have been identified as putative endogenous modulators of neuronal tau phosphorylation and tau exon 10 splicing, respectively (Bottoni et al., 2005; Hébert et al., 2010; Formosa et al., 2012; Rivas et al., 2012).

In conclusion, murine brain signaling pathways modulated by MAM and linked to human neurological disease overlap with those associated with MAM-induced colon cancer. The two disease phenotypes, cancer and neurodegeneration, are mechanistically related; their differential expression may depend respectively on whether or not (as is the case for adult neurons) MAM-exposed cells are able to undergo mitosis, mutagenesis, and uncontrolled cell proliferation (Kisby et al., 2011a). If the foregoing is true, then the lessons learned from cycad-associated ALS-PDC, a prototypical neurodegenerative disorder, should be applied to related diseases of old age.

## Conflict of Interest Statement

The authors declare that the research was conducted in the absence of any commercial or financial relationships that could be construed as a potential conflict of interest.

## References

[B1] Abba M. C. Sun H. Hawkins K. A. Drake J. A. Hu Y. Nunez M. I. Gaddis S. Shi T. Horwath S. Sahin A. Aldaz C. M. (2007). Breast cancer molecular signatures as determined by SAGE: correlation with lymph node status. Mol. Cancer Res. 5, 881–890 10.1158/1541-7786.MCR-07-0055 17855657 PMC4186709

[B2] Adamson R. H. (1989). Induction of hepatocellular carcinoma in nonhuman primates by chemical carcinogens. Cancer Detect. Prev. 14, 215–219 2559797

[B3] Araki W. Kume H. Oda A. Tamaoka A. Kametani F. (2009). IGF-1 promotes beta-amyloid production by a secretase-independent mechanism. Biochem. Biophys. Res. Commun. 380, 11–14 10.1016/j.bbrc.2008.12.171 19167357

[B4] Arvidsson Y. Andersson E. Bergstrom A. Andersson M. K. Altiparmak G. Illerskog A. C. Ahlman H. Lamazhapova D. Nilsson O. (2008). Amyloid precursor-like protein 1 is differentially upregulated in neuroendocrine tumours of the gastrointestinal tract. Endocr. Relat. Cancer 15, 569–581 10.1677/ERC-07-0145 18430897

[B5] Aslam M. I. Patel M. Singh B. Jaemson J. S. Pringle J. H. (2012). MicroRNA manipulation in colorectal cancer cells: from laboratory to clinical application. J. Transl. Med. 10, 128–156 10.1186/1479-5876-10-128 22716183 PMC3539910

[B6] Augustin R. Endres K. Reinhardt S. Kuhn P. H. Lichtenthaler S. F. Hansen J. Wurst W. Trümbach D. (2012). Computational identification and experimental validation of microRNAs binding to the Alzheimer-related gene ADAM10. BMC Med. Genet. 13, 35 10.1186/1471-2350-13-35 22594617 PMC3459808

[B7] Baade P. D. Fritschi L. Freedman D. M. (2007). Mortality due to amyotrophic lateral sclerosis and Parkinson’s disease among melanoma patients. Neuroepidemiology 28, 16–20 10.1159/000097851 17164565

[B8] Bachand A. M. Mundt K. A. Mundt D. J. Montgomery R. R. (2010). Epidemiological studies of formaldehyde exposure and risk of leukemia and nasopharyngeal cancer: a meta-analysis. Crit. Rev. Toxicol. 40, 85–100 10.3109/10408440903352826 20085478

[B9] Bakand S. Winder C. Khalil C. Hayes A. (2005). Toxicity assessment of industrial chemicals and airborne contaminants: transition from in vivo to in vitro test methods: a review. Inhal. Toxicol. 17, 775–787 10.1080/08958370500225240 16195213

[B10] Banack S. A. Johnson H. E. Cheng R. Cox P. A. (2007). Production of the neurotoxin BMAA by a marine cyanobacterium. Mar. Drugs 5, 180–196 10.3390/md504180 18463731 PMC2365698

[B11] Bandrés E. Cubedo E. Agirre X. Malumbres R. Zárate R. Ramirez N. Abajo A. Navarro A. Moreno I. Monzó M. Garvia-Foncillas J. (2006). Identification by real-time PCR of 13 mature microRNAs differentially expressed in colorectal cancer and non-tumoral tissues. Mol. Cancer 5, 29 10.1186/1476-4598-5-29 16854228 PMC1550420

[B12] Bastian I. Tam S. Zhou X. F. Kazenwadel J. Van der Hoek M. Michael M. Z. Gibbins I. Haberberger R. V. (2011). Differential expression of microRNA-1 in dorsal root ganglion neurons. Histochem. Cell Biol. 135, 37–45 10.1007/s00418-010-0772-0 21170745

[B13] Behrens M. I. Lendon C. Roe C. M. (2009). A common biological mechanism in cancer and Alzheimer’s disease. Curr. Alzheimer Res. 6, 196–204 10.2174/156720509788486608 19519301 PMC2810550

[B14] Beltran A. S. Russo A. Lara H. Fan C. Lizardi P. M. Blancafort P. (2011). Suppression of breast tumor growth and metastasis by an engineered transcription factor. PLoS ONE 6, e24595 10.1371/journal.pone.0024595 21931769 PMC3172243

[B15] Bloemen K. Verstraelen S. Van Den Heuvel R. Witters H. Nelissen I. Schoeters G. (2007). The allergic cascade: review of the most important molecules in the asthmatic lung. Immunol. Lett. 113, 6–18 10.1016/j.imlet.2007.07.010 17765979

[B16] Blouet C. Schwartz G. J. (2011). Nutrient-sensing hypothalamic TXNIP links nutrient excess to energy imbalance in mice. J. Neurosci. 31, 6019–6027 10.1523/JNEUROSCI.6498-10.2011 21508227 PMC3100164

[B17] Bobola M. S. Blank A. Berger M. S. Silber J. R. (2007). *O*^6^-Methylguanine-DNA-methyltransferase deficiency in developing brain: implications for brain tumorigenesis. DNA Repair (Amst.) 6, 1127–1133 10.1016/j.dnarep.2007.03.009 17500046 PMC2692685

[B18] Bolós M. Fernandez S. Torres-Aleman I. (2010). Oral administration of a GSK3 inhibitor increases brain insulin-like growth factor I levels. J. Biol. Chem. 285, 17693–17700 10.1074/jbc.M109.096594 20351102 PMC2878533

[B19] Boonen R. A. van Tijn P. Zivkovic D. (2009). Wnt signaling in Alzheimer’s disease: up or down, that is the question. Ageing Res. Rev. 8, 71–82 10.1016/j.arr.2008.11.003 19101658

[B20] Borenstein A. R. Mortimer J. A. Schofield E. Wu Y. Salmon D. P. Gamst A. Olichney J. Thal L. J. Silbert L. Kaye J. Craig U. L. Schellenberg G. D. Galasko D. R. (2007). Cycad exposure and risk of dementia, MCI, and PDC in the Chamorro population of Guam. Neurology 68, 1764–1771 10.1212/01.wnl.0000262027.31623.b2 17515538

[B21] Bottoni A. Piccin D. Tagliati F. Luchin A. Zatelli M. C. degli Uberti E. C. (2005). miR-15a and miR-16-1 down-regulation in pituitary adenomas. J. Cell Physiol. 204, 280–285 10.1002/jcp.20282 15648093

[B22] Brand L. E. Pablo J. Compton A. Hammerschlag N. Mash D. C. (2010). Cyanobacterial blooms and the occurrence of the neurotoxin β-N-methylamino-L-alanine (BMAA) in south Florida aquatic food webs. Harmful Algae 9, 620–635 10.1016/j.hal.2010.05.002 21057660 PMC2968748

[B23] Cai C. Ashktorabm H. Pang X. Zhao Y. Sha W. Liu Y. Gu X. (2010). MicroRNA-211 expression promotes colorectal cancer cell growth in vitro and in vivo by targeting tumor suppressor CHD5. PLoS ONE 7, e29750 10.1371/journal.pone.0029750 22235338 PMC3250477

[B24] Caricasole A. Bakker A. Copani A. Nicoletti F. Gaviraghi G. Terstappen G. C. (2005). Two sides of the same coin: Wnt signaling in neurodegeneration and neuro-oncology. Biosci. Rep. 25, 309–327 10.1007/s10540-005-2893-6 16307379

[B25] Chang K. W. Liu C. J. Chu T. H. Cheng H. W. Hung P. S. Hu W. Y. Lin S. C. (2008). Association between high miR-211 microRNA expression and the poor prognosis of oral carcinoma. J. Dent. Res. 87, 1063–1068 10.1177/154405910808701116 18946016

[B26] Chang S. Wen S. Chen D. Jin P. (2009). Small regulatory RNAs in neurodevelopmental disorders. Hum. Mol. Genet. 18, R18–R26 10.1093/hmg/ddp072 19297398 PMC2657940

[B27] Chen J. Huang X. F. (2009). The signal pathways in azoxymethane-induced colon cancer and preventive implications. Cancer Biol. Ther. 8, 1313–1317 10.1158/1535-7163.TARG-09-C13 19502780

[B28] Chen J. Yang T. Yu H. Sun K. Shi Y. Song W. Bai Y. Wang X. Lou K. Song Y. Zhang Y. Hui R. (2010). A functional variant in the 3′-UTR of angiopoietin-1 might reduce stroke risk by interfering with the binding efficiency of microRNA 211. Hum. Mol. Genet. 19, 2524–2533 10.1093/hmg/ddp479 20378606

[B29] Chen L. Jiang M. Yuan W. Tang H. (2012). miR-17-5p as a novel prognostic marker for hepatocellular carcinoma. J. Invest. Surg. 25, 156–161 10.3109/08941939.2011.618523 22583011

[B30] Chen P. S. Su J. L. Cha S. T. Tarn W. Y. Wang M. Y. Hsu H. C. Lin M. T. Chu C. Y. Hua K. T. Chen C. N. Kuo T. C. Chang K. J. Hsiao M. Chang Y. W. Chen J. S. Yang P. C. Kuo M. L. (2011). miR-107 promotes tumor progression by targeting the let-7 microRNA in mice and humans. J. Clin. Invest. 121, 3442–3455 10.1172/JCI41062 21841313 PMC3163949

[B31] Chin C. L. Curzon P. Schwartz A. J. O’Connor E. M. Rueter L. E. Fox G. B. Day M. Basso A. M. (2011). Structural abnormalities revealed by magnetic resonance imaging in rats prenatally exposed to methylazoxymethanol acetate parallel cerebral pathology in schizophrenia. Synapse 65, 393–403 10.1002/syn.20857 20803618

[B32] Choi K. H. Zepp M. E. Higgs B. W. Weickert C. S. Webster M. J. (2009). Expression profiles of schizophrenia susceptibility genes during human prefrontal cortical development. J. Psychiatry Neurosci. 34, 450–458 19949721 PMC2783436

[B33] Clifford M. A. Kanwal J. K. Dzakpasu R. Donoghue M. J. (2011). EphA4 expression promotes network activity and spine maturation in cortical neuronal cultures. Neural Dev. 6, 21 10.1186/1749-8104-6-21 21542907 PMC3100241

[B34] Cogswell J. P. Ward J. Taylor I. A. Waters M. Shi Y. Cannon B. Kelnar K. Kemppainen J. Brown D. Chen C. Prinjha R. K. Richardson J. C. Saunders A. M. Roses A. D. Richards C. A. (2008). Identification if miRNA changes in Alzheimer’s disease brain and CSF yields putative biomarkers and insights into disease pathways. J. Alzheimers Dis. 14, 27–41 18525125 10.3233/jad-2008-14103

[B35] Cooper T. A. Wan L. Drefuss G. (2009). RNA and disease. Cell 136, 777–793 10.1016/j.cell.2009.02.011 19239895 PMC2866189

[B36] Crawford M. Batte K. Yu L. Wu X. Nuovo G. J. Marsh C. B. Otterson G. A. Nana-Sinkam S. P. (2009). MicroRNA 133B targets prosurvival molecules MCL-1 and BCL2L2 in lung cancer. Biochem. Biophys. Res. Commun. 388, 483–489 10.1016/j.bbrc.2009.07.143 19654003 PMC2824514

[B37] Cuatrecasas M. Santamaria G. Velasco M. Camacho E. Hernandez L. Sanchez M. Orrit C. Murcia C. Cardesa A. Campo E. Fernandez P. L. (2006). ATM gene expression and angiogenesis in infiltrating breast carcinomas. Histol. Histopathol. 21, 149–156 16329039 10.14670/HH-21.149

[B38] Datta J. Kutay H. Nasser M. W. Nuovo G. J. Wang B. Majumder S. Liu C. G. Volinia S. Croce C. M. Schmittgen T. D. Ghoshal K. Jacob S. T. (2008). Methylation mediated silencing of microRNA-1 gene and its role in hepatocellular carcinogenesis. Cancer Res. 68, 5049–5058 10.1158/0008-5472.CAN-07-6655 18593903 PMC2562630

[B39] Davidson L. A. Wang N. Shah M. S. Lupton J. R. Ivanov I. Chapkin R. S. (2009). n-3 Polyunsaturated fatty acids modulate carcinogen-directed non-coding microRNA signatures in rat colon. Carcinogenesis 30, 2077–2084 10.1093/carcin/bgp245 19825969 PMC2792315

[B40] De La Monte S. M. (2009). Insulin resistance and Alzheimer’s disease. BMB Rep. 42, 475–481 10.5483/BMBRep.2009.42.8.475 19712582 PMC4600067

[B41] de Sousa Andrade L. N. Nathanson J. L. Yeo G. W. Mencj C. F. Muotri A. R. (2012). Evidence for premature aging due to oxidative stress in iPSCs from Cockayne syndrome. Hum. Mol. Genet. 21, 3825–3834 10.1093/hmg/dds211 22661500 PMC3412382

[B42] de Strooper B. (2010). Cancer and neurodegeneration meet. EMBO Mol. Med. 2, 245–246 10.1002/emmm.201000078 20583009 PMC3377326

[B43] Dhanasekaran S. M. Barrette T. R. Ghosh D. Shah R. Varambally S. Kurachi K. Pienta K. J. Rubin M. A. Chinnaiyan A. M. (2001). Delineation of prognostic biomarkers in prostate cancer. Nature 412, 822–826 10.1038/35090585 11518967

[B44] Du L. Pertsemlidis A. (2011). Cancer and neurodegenerative disorders: pathogenic convergence through microRNA regulation. J. Mol. Cell Biol. 3, 176–180 10.1093/jmcb/mjq058 21278200 PMC3104012

[B45] Featherstone R. E. Rizos Z. Nobrega J. N. Kapur S. Fletcher P. J. (2007). Gestational methylazoxymethanol acetate treatment impairs select cognitive functions: parallels to schizophrenia. Neuropsychopharmacology 32, 483–492 10.1038/sj.npp.1301223 17035930

[B46] Feng W. Feng Y. (2011). MicroRNAs in neural development and brain diseases. Sci. China Life Sci. 54, 1103–1112 10.1007/s11427-011-4212-8 22227902

[B47] Formosa A. Lena A. M. Markert E. K. Cortelli S. Miano R. Mauriello A. Croce N. Vandesompele J. Mestdagh P. Finazzi-Agrò E. Levine A. J. Melino G. Bernadini S. Candi E. (2012). DNA methylation silences miR-132 in prostate cancer. Oncogene [Epub ahead of print] 10.1038/onc.2012.14 22310291

[B48] Gallo V. Bueno-De-Mesquita H. B. Vermeulen R. Andersen P. M. Kyrozis A. Linseisen J. Kaaks R. Allen N. E. Roddam A. W. Boshuizen H. C. Peeters P. H. Palli D. Mattiello A. Sieri S. Tumino R. Jiménez-Martín J. M. Díaz M. J. Suarez L. R. Trichopoulou A. Agudo A. Arriola L. Barricante-Gurrea A. Bingham S. Khaw K. T. Manjer J. Lindkvist B. Overvad K. Bach F. W. Tjønneland A. Olsen A. Bergmann M. M. Boeing H. Clavel-Chapelon F. Lund E. Hallmans G. Middleton L. Vineis P. Riboli E. (2009). Smoking and risk for ALS. Ann. Neurol. 65, 378–385 10.1002/ana.21653 19399866

[B49] Gascon E. Gao F. B. (2012). Cause or effect: misregulation of microRNA pathways in neurodegeneration. Front. Neurosci. 6:48 10.3389/fnins.2012.00048 22509148 PMC3321503

[B50] Gatti R. A. Berkel I. Boder E. Braedt G. Charmley P. Concannon P. Ersoy F. Foroud T. Jaspers N. G. Lange K. (1988). Localization of an ataxia-telangiectasia gene to chromosome 11q22-23. Nature 336, 577–580 10.1038/336577a0 3200306

[B51] Gobe G. C. (1994). Apoptosis in brain and gut tissue of mice fed a seed preparation of the cycad *Lepidozamia peroffskyana*. Biochem. Biophys. Res. Commun. 30, 327–333 10.1006/bbrc.1994.2668 7999044

[B52] Gong M. Ma J. Li M. Zhou M. Hock J. M. Yu X. (2012). MicroRNA-204 critically regulates carcinogenesis in malignant peripheral nerve sheath tumors. Neuro. Oncol. 14, 1007–1017 10.1093/neuonc/nos124 22718995 PMC3408257

[B53] Grafstrom R. C. Curren R. D. Yang L. L. Harris C. C. (1985). Genotoxicity of formaldehyde in cultured human bronchial fibroblasts. Science 228, 89–91 10.1126/science.3975633 3975633

[B54] Gutierrez-Aguilar R. Kim D. H. Woods S. C. Seeley R. J. (2012). Expression of new loci associated with obesity in diet-induced obese rats: from genetics to physiology. Obesity (Silver Spring) 20, 306–312 10.1038/oby.2011.236 21779089

[B55] Haller F. von Heydebreck A. Zhang J. D. Gunawan B. Langer C. Ramadori G. Wiemann S. Sahin O. (2010). Localization- and mutation-dependent microRNA (miRNA) expression signatures in gastrointestinal stromal tumours (GISTs), with a cluster of co-expressed miRNAs located at 14q32.31. J. Pathol. 220, 71–86 10.1002/path.2610 19768731

[B56] Han C. Wan G. Langley R. R. Zhang X. Lu X. (2012). Crosstalk between DNA damage response pathways and miRNAs. Cell Mol. Life Sci. 69, 2895–2906 10.1007/s00018-012-0959-8 22430204 PMC11115143

[B57] Han L. Wen Z. Lynn R. C. Baudet M. L. Holt C. E. Sasaki Y. Bassell G. J. Zheng J. Q. (2011). Regulation of chemotropic guidance of nerve growth cones by microRNA. Mol. Brain 4, 40 10.1186/1756-6606-4-32 22051374 PMC3217933

[B58] Hansel D. E. Rahman A. Wehner S. Herzog S. Yeo C. J. Maitra A. (2003). Increased expression and processing of the Alzheimer amyloid precursor protein in pancreatic cancer may influence cellular proliferation. Cancer Res. 63, 7032–7037 14612490

[B59] Haramati S. Chapnik E. Sztainberg Y. Eilam R. Zwang R. Gershoni N. McGlinn E. Heiser P. W. Wills A. M. Wirguin I. Rubin L. L. Misawa H. Tabin C. J. Brown R. Jr. Chen A. Hornstein E. (2010). miRNA malfunction causes spinal motor neuron disease. Proc. Natl. Acad. Sci. U.S.A. 107, 13111–13116 10.1073/pnas.1006151107 20616011 PMC2919953

[B60] He R. Q. Lu J. Miao J. Y. (2010). Formaldehyde stress. Sci. China Life Sci. 53, 1399–1404 10.1007/s11427-010-3105-6 21181340

[B61] Hébert S. S. Horré K. Nicolaï L. Bergmans B. Papadopoulou A. S. Delacourte A. De Strooper B. (2009). MicroRNA regulation of Alzheimer’s amyloid precursor protein expression. Neurobiol. Dis. 33, 422–428 10.1016/j.nbd.2008.11.009 19110058

[B62] Hébert S. S. Papadoupolou A. S. Smith P. Galas M. C. Planel E. Silahtaroglu A. N. Sergeant N. Buée L. De Strooper B. (2010). Genetic ablation of Dicer in adult forebrain neurons results in abnormal tau hyperphosphorylation and neurodegeneration. Hum. Mol. Genet. 19, 3959–3969 10.1093/hmg/ddq311 20660113

[B63] Hirono I. Shibuya C. (1967). Induction of a neurological disorder by cycasin in mice. Nature 216, 1311–1312 10.1038/2161311a0 6080057

[B64] Hossain A. Kuto M. T. Sunders G. F. (2006). Mir-17-5p regulates breast cancer cell proliferation by inhibiting translation of AIB1 mRNA. Mol. Cell Biol. 26, 8191–8201, 2006. 10.1128/MCB.00242-06 16940181 PMC1636750

[B65] Hradetzky E. Sanderson T. M. Tsang T. M. Sherwood J. L. Fitzjohn S. M. Lakics V. Malik N. Schoeffmann S. O’Neill M. J. Cheng T. M. Harris L. W. Rahmoune H. Guest P. C. Sher E. Collingridge G. Sl. Holmes E. Tricklebank M. D. Bahn S. (2012). The methylazoxymethanol acetate (MAM-E17) rat model: molecular and functional effects in the hippocampus. Neuropsychopharmacology 37, 364–377 10.1038/npp.2011.219 21956444 PMC3242314

[B66] Hu H. Chen D. Li X. Yang K. Wang H. Wu W. (2010). miR-133b regulates the MET proto-oncogene and inhibits the growth of colorectal cancer cells in vitro and in vivo. Cancer Biol. Ther. 10, 190–197 10.4161/cbt.10.2.12186 20505319

[B67] Hudson R. S. Yi M. Esposito D. Watkins S. K. Hurwitz A. A. Yfantis H. G. Lee D. H. Borin J. F. Naslund M. J. Alexander R. B. Dorsey T. H. Stephens R. M. Croce C. M. Ambs S. (2012). Micro-RNA-1 is a candidate tumor suppressor and prognostic marker in human prostate cancer. Nucleic Acids Res. 40, 3689–3703 10.1093/nar/gkr1222 22210864 PMC3333883

[B68] Ikeda Y. Tanji E. Makino N. Kawata S. Furukawa T. (2012). MicroRNAs associated with mitogen-activated protein kinase in human pancreatic cancer. Mol. Cancer Res. 10, 259–269 10.1158/1541-7786.MCR-11-0035 22188669

[B69] Inoue E. Deguchi-Tawarada M. Togawa A. Matsui C. Arita K. Katahira-Tayama S. Sato T. Yamauchi E. Oda Y. Takai Y. (2009). Synaptic activity prompts gamma-secretase-mediated cleavage of EphA4 and dendritic spine formation. J. Cell Biol. 185, 551–564 10.1083/jcb.200809151 19414612 PMC2700400

[B70] International Agency for Research on Cancer (IARC) (2006). IARC Monographs on the Evaluation of Carcinogenic Risks to Humans: Formaldehyde, 2-Butoxyethanol and 1-tert-Butoxypropan-2-ol. Lyon: International Agency for Research on Cancer 88 PMC4781641 17366697

[B71] Iorio M. V. Ferracin M. Liu C. G. Veronese A. Spizzo R. Sabbioni S. Magri E. Pedriali M. Fabbri M. Campiglio M. Menard S. Palazzo J. P. Rosenberg A. Musiani P. Volinia S. Nenci I. Calin G. A. Querzoli P. Negrini M. Croce M. (2005). MicroRNA gene expression deregulation in human breast cancer. Cancer Res. 65, 7065–7070 10.1158/0008-5472.CAN-05-1783 16103053

[B72] Izzotti A. Calin G. A. Arrigo P. Steele V. E. Croce C. M. De Flora S. (2009). Downregulation of microRNA expression in the lungs of rats exposed to cigarette smoke. FASEB J. 23, 806–812 10.1096/fj.09-135251 18952709 PMC2653990

[B73] Jaspers I. Flescher E. Chen L. C. (1997). Ozone-induced IL-8 expression and transcription factor binding in respiratory epithelial cells. Am. J. Physiol. Lung Cell. Mol. Physiol. 272, L504–L511 10.1152/ajplung.1997.272.3.L504 9124608

[B74] Jimenez-Mateos E. M. Engel T. Merino-Serrais P. McKiernan R. C. Tanaka K. Mouri G. Sano T. O’Tuathaigh C. Waddington J. L. Prenter S. Delanty N. Farrell M. A. O’Brien D. F. Conroy R. M. Stallings R. L. Defelipe J. Henshall D. C. (2012). Silencing microRNA-134 produces neuroprotective and prolonged seizure-suppressive effects. Nat. Med. 18, 1087–1094 10.1038/nm.2834 22683779 PMC3438344

[B75] Jones M. Z. Gardner E. (1976). Pathogenesis of methylazoxymethanol-induced lesions in the postnatal mouse cerebellum. J. Neuropathol. Exp. Neurol. 35, 413–444 10.1097/00005072-197607000-00004 932788

[B76] Jorens P. Damme J. V. Backer W. D. Bossaert L. DeJongh R. F. Herman A. Rampart M. (1992). Interleukin 8 (IL-8) in the bronchoalveolar lavage fluid from patients with the adult respiratory distress syndrome (ARDS) and patients at risk for ARDS. Cytokine 4, 592–597 10.1016/1043-4666(92)90025-M 1292643

[B77] Kano M. Seki N. Kikkawa N. Fujimura L. Hoshino I. Akutsu Y. Chiyomaru T. Enokida H. Nakagawa M. Matsubara H. (2010). miR-145, miR-133a and miR-133b: tumor-suppressive miRNAs target FSCN1 in esophageal squamous cell carcinoma. Int. J. Cancer 127, 2804–2814 10.1002/ijc.25284 21351259

[B78] Kantrowitz J. Javitt D. C. (2012). Glutamatergic transmission in schizophrenia: from basic research to clinical practice. Curr. Opin. Psychiatry 25, 96–102 22297716 10.1097/YCO.0b013e32835035b2 PMC5224527

[B79] Karin M. Greten F. R. (2005). NF-κB: linking inflammation and immunity to cancer development and progression. Nat. Rev. Immunol. 5, 749–759 10.1038/nri1703 16175180

[B80] Kawaai K. Tominaga-Yoshino K. Urakubo T. Taniguchi N. Kondoh Y. Tashiro H. Ogura A. Tashiro T. (2010). Analysis of gene expression changes associated with long-lasting synaptic enhancement in hippocampal slice cultures after repetitive exposures to glutamate. J. Neurosci. Res. 88, 2911–2922 20568283 10.1002/jnr.22457

[B81] Kawahara Y. (2010). Implications of microRNA dysfunction in the pathogenesis of ALS. Rinsho Shinkeigaku 50, 979–981 10.5692/clinicalneurol.50.979 21921534

[B82] Kawahara Y. Mieda-Sato A. (2012). TDP-43 promotes microRNA biogenesis as a component of the Drosha and Dicer complexes. Proc. Natl. Acad. Sci. U.S.A. 109, 3347–3352 10.1073/pnas.1112427109 22323604 PMC3295278

[B83] Kawakami K. Enokida H. Chiyomaru T. Tatarano S. Yoshino H. Kagara I. Gotanda T. Tachiwada T. Nishiyama K. Nohata N. Seki N. Nakagawa M. (2012). The functional significance of miR-1 and miR-133a in renal cell carcinoma. Eur. J. Cancer 48, 827–836 10.1016/S0959-8049(12)71662-3 21745735

[B84] Kerns W. D. Pavkov K. L. Donofrio D. J. Gralla E. J. Swenberg J. A. (1983). Carcinogenicity of formaldehyde in rats and mice after long-term inhalation exposure. Cancer Res. 43, 4382–4392 6871871

[B85] Khudayberdiev S. Fiore R. Schratt G. (2009). MicroRNA as modulators of neuronal responses. Commun. Integr. Biol. 2, 411–413 10.4161/cib.2.5.8834 19907703 PMC2775236

[B86] Kim J. Inoue K. Ishii J. Vanti W. B. Voronov S. V. Murchison E. Hannon G. Abeliovich A. (2007). A MicroRNA feedback circuit in midbrain dopamine neurons. Science 317, 1220–1224 10.1126/science.1143993 17761882 PMC2782470

[B87] Kisby G. E. Ellison M. Spencer P. S. (1992a). Content of the neurotoxins cycasin (methylazoxymethanol β-D-glucoside) and BMAA (β-*N*-methylamino-L-alanine) in cycad flour prepared by Guam Chamorros. Neurology 42, 1336–1340 10.1212/WNL.42.7.1336 1620343

[B88] Kisby G. E. Nottingham V. Kayton R. Roy D. N. Spencer P. S. (1992b). Brain metabolism of β-*N*-methylamino-L-alanine (BMAA) and protection of excitotoxicity by GABA-uptake inhibitors. Soc. Neurosci. Abstr. 18, 82

[B89] Kisby G. E. Ross S. M. Spencer P. S. Gold B. G. Nunn P. B. Roy D. N. (1992c). Cycasin and BMAA: candidate neurotoxins for Western Pacific amyotrophic lateral sclerosis/Parkinsonism-dementia complex. Neurodegeneration 1, 73–82

[B90] Kisby G. E. Fry R. C. Lasarev M. R. Bammler T. K. Beyer R. P. Churchwell M. Doerge D. R. Meira L. B. Palmer V. S. Ramos-Crawford A. L. Ren X. Sullivan R. C. Kavanagh T. J. Samson L. D. Zarbl H. Spencer P. S. (2011a). The cycad genotoxin MAM modulates brain cellular pathways involved in neurodegenerative disease and cancer in a DNA damage-linked manner. PLoS ONE 6, e20911 10.1371/journal.pone.0020911 21731631 PMC3121718

[B91] Kisby G. Palmer V. Lasarev M. Fry R. Iordanov M. Magun E. Samson L. Spencer P. S. (2011b) Does the cycad genotoxin MAM implicated in Guam ALS-PDC induce disease-relevant changes in mouse brain that includes olfaction? Commun. Integr. Biol. 4, 731–734 22446540 10.4161/cib.17603 PMC3306344

[B92] Kisby G. E. Renslow P. Ryan A. Beam M. Woltjer R. (2011c). The cycad genotoxin methylazoxymethanol (MAM) induces brain tissue DNA damage and accelerates tau pathology in htau mice. Soc. Neurosci Abstr. Available at: http://www.sfn.org/index.aspx?pagename=abstracts_ampublications&section=publications

[B93] Kisby G. E. Kabel H. Hugon J. Spencer P. (1999). Damage and repair of nerve cell DNA in toxic stress. Drug Metab. Rev. 31, 589–618 10.1081/DMR-100101937 10461542

[B94] Kisby G. E. Olivas A. Park T. Churchwell M. Doerge D. Samson L. D. Gerson S. L. Turker M. S. (2009). DNA repair modulates the vulnerability of the developing brain to alkylating agents. DNA Repair (Amst.) 8, 400–412 10.1016/j.dnarep.2008.12.002 19162564 PMC2692311

[B95] Kisby G. E. Spencer P. S. (2011). Is neurodegenerative disease a long-latency response to early-life genotoxin exposure? Int. J. Environ. Res. Public Health 8, 3889–3921 10.3390/ijerph8103889 22073019 PMC3210588

[B96] Kisby G. E. Eizirik D. Sweatt C. Spencer P. S. (1995). Reactive oxygen species produced by the cycad toxin methylazoxymethanol, a candidate etiological factor for western Pacific ALS/P-D. J. Cell Biochem. 21B, 99

[B97] Ko S. Y. Chang K. W. Lin S. C. Hsu H. C. Liu T. Y. (2007). The repressive effect of green tea ingredients on amyloid precursor protein (APP) expression in oral carcinoma cells *in vitro* and *in vivo*. Cancer Lett. 245, 81–89 10.1016/j.canlet.2005.10.019 16458426

[B98] Kojima S. Chiyomaru T. Kawakami K. Yoshino H. Enokida H. Nohata N. Fuse M. Ichikawa T. Naya Y. Nakagawa M. Seki N. (2012). Tumour suppressors miR-1 and miR-133a target the oncogenic function of purine nucleoside phosphorylase (PNP) in prostate cancer. Br. J. Cancer 106, 405–413 10.1038/bjc.2011.462 22068816 PMC3261671

[B99] Krause K. Karger S. Sheu S. Y. Aigner T. Kursawe R. Gimm O. Schmid K. W. Dralle H. Fuhrer D. (2008). Evidence for a role of amyloid precursor protein in thyroid carcinogenesis. J. Endocrinol. 198, 291–299 10.1677/JOE-08-0005 18480379

[B100] Kumar K. R. Djarmati-Westenberger A. Grünewald A. (2011). Genetics of Parkinson’s disease. Semin. Neurol. 31, 433–440 10.1055/s-0031-1277986 22266881

[B101] La Maestra S. Kisby G. E. Micale R. T. Johnson J. Kow Y. W. Bao G. Sheppard C. Stanfield S. Tran H. Woltjer R. L. D’Agostini F. Steele V. E. De Flora S. (2011). Cigarette smoke induces DNA damage and alters base-excision repair and tau levels in the brain of neonatal mice. Toxicol. Sci. 123, 471–479 10.1093/toxsci/kfr187 21778470 PMC3179679

[B102] Lacqueur G. L. Spatz M. (1973). “Transplacental induction of tumors and malformations in rats with cycasin and methylazoxymethanol,” in Transplacental Carcinogenesis, ed. Tomatis L. Mohr I. (Geneva: International Agency for Research on Cancer Press), 59–64

[B103] Lafarga M. Lerga A. Andres M. A. Polanco J. L. Calle E. Berciano M. T. (1997). Apoptosis induced by methylazoxymethanol in developing rat cerebellum: organization of the cell nucleus and its relationship to DNA and rRNA regulation. Cell Tissue Res. 289, 25–38 10.1007/s004410050849 9182598

[B104] LaFerla F. M. Green K. N. Oddo S. (2007). Intracellular amyloid-beta in Alzheimer’s disease. Nat. Rev. Neurosci. 8, 499–509 10.1038/nrn2168 17551515

[B105] Lam E. K. Wang X. Shin V. Y. Zhang S. Morrison H. Sun J. Ng E. K. Yu J. Jin H. (2011). A microRNA contribution to aberrant Ras activation in gastric cancer. Am. J. Transl. Res. 3, 209–218 21416062 PMC3056566

[B106] Law P. T. Wong N. (2011). Emerging roles of microRNA in the intracellular signaling networks of hepatocellular carcinoma. J. Gastoenterol. Hepatol. 26, 437–449 10.1111/j.1440-1746.2010.06512.x 21332540

[B107] Lee E. S. Chen H. Hardman C. Simm A. Charlton C. (2008). Excessive *S*-adenosyl-L-methionine-dependent methylation increases levels of methanol, formaldehyde and formic acid in rat brain striatal homogenates: possible role in *S*-adenosyl-L-methionine-induced Parkinson’s disease-like disorders. Life Sci. 83, 821–827 10.1016/j.lfs.2008.07.014 18930743 PMC2885904

[B108] Lee K. H. Chen Y. L. Yeh S. D. Hsiao M. Lin J. T. Goan Y. G. Lu P. J. (2009). MicroRNA-330 acts as tumor suppressor and induces apoptosis of prostate cancer cells through E2F1-mediated suppression of Akt phosphorylation. Oncogene 28, 3360–3370 10.1038/onc.2009.192 19597470

[B109] Lee Y. Yang X. Huang Y. Fan H. Zhang Q. Wu Y. Li J. Hasina R. Cheng C. Lingen M. W. Gerstein M. B. Weichselnbaum R. R. Xing X. R. Lussier Y. A. (2010). Network modeling identifies molecular functions targeted by miR-204 to suppress head and neck tumor metastasis. PLoS Comput. Biol. 6, e1000730 10.1371/journal.pcbi.1000730 20369013 PMC2848541

[B110] Lee Y. S. Dutta A. (2007). The tumor suppressor microRNA let-7 represses the HMGA2 oncogene. Genes Dev. 21, 1025–1030 10.1101/gad.1518407 17437991 PMC1855228

[B111] Leone V. D’Angelo D. Rubio I. de Freitas P. M. Federico A. Colamaio M. Pallante P. Medeiros-Neto G. Fusco A. (2011). MiR-1 is tumor suppressor in thyroid carcinogenesis targeting CCND2, CXCR4, and SDF-1alpha. J. Clin. Endocrinol. Metab. 96, e1388–e1398 10.1210/jc.2011-0345 21752897

[B112] Leucci E. Zriwil A. Gregersen L. H. Jensen K. T. Obad S. Bellan C. Leoncini L. Kauppinen S. Lund A. H. (2012). Inhibition of miR-9 de-represses HuR and DICER and impairs Hodgkin lymphoma tumor outgrowth *in vivo*. Oncogene [Epub ahead of print]. 10.1038/onc.2012.15 22310293

[B113] Leucht C. Stigloher C. Wizenmann A. Klafke R. Folchert A. Bally-Cuif L. (2008). MicroRNA-9 directs late-organizer activity of the midbrain-hindbrain boundary. Nat. Neurosci. 11, 641–648 10.1038/nn.2115 18454145

[B114] Levine S. Saltzman A. Levy E. Ginsberg S. D. (2009). Systemic pathology in aged mouse models of Down’s syndrome and Alzheimer’s disease. Exp. Mol. Pathol. 86, 18–22 10.1016/j.yexmp.2008.10.006 19041304 PMC2659493

[B115] Levy C. Khaled M. Iliopoulos D. Janas M. M. Schubert S. Pinner S. Chen P. H. Li S. Fletcher A. L. Yokoyama S. Scott K. L. Garrarway L. A. Song J. S. Granter S. R. Turley S. J. Fisher D. E. Novina C. D. (2010). Intronic miR-211 assumes the tumor suppressive function of its host gene in melanoma. Mol. Cell 40, 841–849 10.1016/j.molcel.2010.11.020 21109473 PMC3004467

[B116] Lewis C. A. Manning J. Rossi F. Krieger C. (2012). The neuroinflammatory response in ALS: the roles of microglia and T cells. Neurol. Res. Int. 2012, 803701 22666587 10.1155/2012/803701 PMC3362167

[B117] Li G. Y. Lee H. Y. Shin H. S. Kim H. Y. Lim C. H. Lee B. H. (2007). Identification of gene markers for formaldehyde exposure in humans. Environ. Health Perspect. 115, 1460–1466 10.1289/ehp.10325 17938736 PMC2022670

[B118] Li H. G. Luna C. Qiu J. Epstein D. L. Gonzalez P. (2011). Role of miR-204 in the regulation of apoptosis, endoplasmic reticulum stress response, and inflammation in human trabecular meshwork cells. Invest. Ophthalmol. Vis. Sci. 52, 2999–3007 10.1167/iovs.10-6868 21282569 PMC3109013

[B119] Ligorio M. Izzotti A. Pulliero A. Arrigo P. (2011). Mutagens interfere with microRNA maturation by inhibiting DICER. An in silico biology analysis. Mutation Res. 717, 116–128 10.1016/j.mrfmmm.2011.07.020 21889945

[B120] Liu D. Z. Ander B. P. Tian Y. Stamova B. Jickling G. C. Davis R. R. Sharp F. R. (2012). Integrated analysis of mRNA and microRNA expression in mature neurons, neural progenitor cells and neuroblastoma cells. Gene 495, 120–127 10.1016/j.gene.2011.12.041 22244746

[B121] Liu Y. Huang T. Zhao X. Cheng L. (2011). MicroRNAs modulate the Wnt signaling pathway through targeting its inhibitors. Biochem. Biophys. Res. Commun. 408, 259–264 10.1016/j.bbrc.2011.04.009 21501592

[B122] Lodge D. J. Grace A. A. (2009). Gestational methylazoxymethanol acetate administration: a developmental disruption model of schizophrenia. Behav. Brain Res. 203, 306–312 10.1016/j.bbr.2009.01.031 19716984 PMC2736136

[B123] Lovell K. L. Jones M. Z. (1980). Partial external germinal layer regeneration in the cerebellum following methylazoxymethanol administration. Effects on Purkinje cell dendritic spines. J. Neuropathol. Exp. Neurol. 39, 541–548 10.1097/00005072-198009000-00003 7217999

[B124] Lu J. Getz G. Miska E. A. Alvarez-Saavedra E. Lamb J. Peck D. Sweet-Cordero A. Ebert B. L. Mak R. H. Ferrando A. A. Downing J. R. Jacks T. Horvitz H. R. Golub T. R. (2005). MicroRNA expression profiles classify human cancers. Nature 435, 834–838 10.1038/nature03702 15944708

[B125] Lujambio A. Esteller M. (2007). CpG island hypermethylation of tumor suppressor microRNAs in human cancer. Cell Cycle 6, 1455–1459 10.4161/cc.6.12.4408 17581274

[B126] Ma L. Teruya-Feldstein J. Weinberg R. A. (2007). Tumour invasion and metastasis initiated by microRNA-10b in breast cancer. Nature 449, 682–688 10.1038/nature06174 17898713

[B127] Marcucci G. Radmacher M. Mrózek K. Bloomfield C. (2009). MicroRNA expression in acute myeloid leukemia. Curr. Hematol. Malig. Rep. 4, 83–88 10.1007/s11899-009-0012-7 20425419

[B128] Margis R. Margis R. Rieder C. R. (2011). Identification of blood microRNAs associated to Parkinson’s disease. J. Biotechnol. 152, 96–101 10.1016/j.jbiotec.2011.01.023 21295623

[B129] Marsh G. M. Youk A. O. (2004). Reevaluation of mortality risks from leukemia in the formaldehyde cohort study of the National Cancer Institute. Regul. Toxicol. Pharmacol. 40, 113–124 10.1016/j.yrtph.2004.05.012 15450715

[B130] Martel M. A. Soriano F. X. Baxter P. Rickman C. Duncan R. Wyllie D. J. Hardingham G. E. (2009). Inhibiting pro-death NMDA receptor signaling dependent on the NR2 PDZ ligand may not affect synaptic function or synaptic NMDA receptor signaling to gene expression. Channels (Austin) 3, 12–15 10.4161/chan.3.1.7864 19221512

[B131] Martin J. J. (2008). DNA damage and repair: relevance to mechanisms of neurodegeneration. J. Neuropathol. Exp. Neurol. 67, 377–387 10.1097/NEN.0b013e31816ff780 18431258 PMC2474726

[B132] Matsumoto H. Higa H. H. (1966). Studies on methylazoxymethanol, the aglycone of cycasin: methylation of nucleic acids *in vitro*. Biochem. J. 98, 20c–22c 5941330 10.1042/bj0980020c PMC1264889

[B133] McClintock D. Zhuo H. Wickersham N. Matthay M. Ware L. (2008). Biomarkers of inflammation, coagulation and fibrinolysis predict mortality in acute lung injury. Critical Care 12, R41 10.1186/cc6262 18358078 PMC2447583

[B134] McGeer P. L. McGeer E. G. (1998). Mechanisms of cell death in Alzheimer’s disease – immunopathology. J. Neural Transm. Suppl. 54, 159–166 9850924 10.1007/978-3-7091-7508-8_15

[B135] McGeer P. L. McGeer E. G. (2001). Inflammation, autotoxicity and Alzheimer disease. Neurobiol. Aging 22, 799–809 10.1016/S0197-4580(01)00288-3 11754986

[B136] McGeer P. L. Steele J. C. (2011). The ALS/PDC syndrome of Guam: potential biomarkers for an enigmatic disorder. Progr. Neurobiol. 95, 663–669 10.1016/j.pneurobio.2011.04.001 21527311

[B137] Mehdi S. J. Alam M. S. Batra S. Rizvi M. M. (2011). Allelic loss of 6q25-27, the PARKIN tumor suppressor gene locus, in cervical carcinoma. Med. Oncol. 28, 1520–1526 10.1007/s12032-010-9633-x 20652448

[B138] Merker R. J. Graham P. Cressman V. Kisby G. E. Levin M. Moore H. (2009). Abnormal neonatal and adult gene expression patterns in the hippocampus and prefrontal cortex of offspring of rat dams exposed to MAM on embryonic day 17. Soc. Neurosci. Abstr. Available at: http://www.sfn.org/index.aspx?pagename=abstracts_ampublications&section=publications

[B139] Migliore C. Martin V. Leoni V. P. Restivo A. Atzori L. Petrelli A. Isella C. Zorcolo L. Sarotto I. Casula G. Comoglio P. M. Columbano A. Giordano S. (2012). MiR-1 downregulation cooperates with MACC1 in promoting MET overexpression in human colon cancer. Clin. Cancer Res. 18, 737–747 10.1158/1078-0432.CCR-11-1699 22179665

[B140] Mikhaylova O. Straton Y. Hall D. Kellner E. Ehmer B. Drew A. F. Gallo C. A. Plas D. R. Biesiada J. Meller J. Czyzyk-Krzeska M. F. (2012). VHL-regulated MiR-204 suppresses tumor growth through inhibition of LC3B-mediated autophagy in renal clear cell carcinoma. Cancer Cell 21, 532–546 10.1016/j.ccr.2012.02.019 22516261 PMC3331999

[B141] Ming G. Song H. (2005). Adult neurogenesis in the mammalian central nervous system. Annu. Rev. Neurosci. 28, 223–250 10.1146/annurev.neuro.28.051804.101459 16022595

[B142] Minones-Moyano E. Porta S. Escaramis G. Rabionet R. Iraola S. Kagerbauer B. Espinosa-Parilla Y. Ferrer I. Estivill X. Marti E. (2011). MicroRNA profiling of Parkinson’s disease brains identifies early downregulation of miR-34b/c which modulate mitochondrial function. Hum. Mol. Genet. 20, 3067–3078 10.1093/hmg/ddr210 21558425

[B143] Mishima Y. Stahlhut C. Giraldez A. J. (2007). miR-1-2 gets to the heart of the matter. Cell 129, 247–249 10.1016/j.cell.2007.04.008 17448987

[B144] Mondo K. Hammerschlag N. Basile M. Pablo J. Banack S. A. Mash D. C. (2012). Cyanobacterial neurotoxin β-*N*-methylamino-L-alanine (BMAA) in shark fins. Mar. Drugs 10, 509–520 10.3390/md10020509 22412816 PMC3297012

[B145] Mouradian M. M. (2012). MicroRNAs and Parkinson’s disease. Neurobiol. Dis. 46, 279–284 10.1016/j.nbd.2011.12.046 22245218

[B146] Nagasawa H. T. Shirota F. N. Matsumoto H. (1972). Decomposition of methylazoxymethanol, the aglycone of cycasin, in D_2_O. Nature 235, 234–235 10.1038/236234a0 4553643

[B147] Nagata Y. Matsumoto H. (1969). Studies on methylazoxymethanol: methylation of nucleic acid in the fetal brain. Proc. Soc. Exp. Biol. 122, 383–385 5344864 10.3181/00379727-132-34220

[B148] Nambotin S. B. Wands J. R. Kim M. (2011). Points of therapeutic intervention along the Wnt signaling pathway in hepatocellular carcinoma. Anticancer Agents Med. Chem. 11, 549–559 21554202 10.2174/187152011796011019

[B149] National Toxicology Program (2010). Final report on carcinogens: background document for formaldehyde. Rep. Carcinog. Backgr. Doc. (10–5981), i–512 20737003

[B150] Negrini M. Gramantieri L. Sabbioni S. Croce C. M. (2011). MicroRNA involvement in hepatocellular carcinoma. Anticancer Agents Med. Chem. 11, 500–521 21554203 10.2174/187152011796011037

[B151] Nejak-Bowen K. N. Monga S. P. (2011). β-Catenin signaling, liver regeneration and hepatocellular cancer: sorting the good from the bad. Semin. Cancer Biol. 21, 44–58 10.1016/j.semcancer.2010.12.010 21182948 PMC3050081

[B152] Nigro N. D. (1985). Animal model for colorectal cancer. Prog. Clin. Biol. Res. 186, 161–173 4034597

[B153] Nohata N. Hanazawa T. Enokida H. Seki N. (2012). microRNA-1/133a and microRNA-206/133b clusters: dysregulation and functional roles in human cancers. Oncotarget 3, 9–21 22308266 10.18632/oncotarget.424 PMC3292888

[B154] O’Carroll D. Schaefer A. (2012). General principals of miRNA biogenesis and regulation in the brain. Neuropsychopharmacology [Epub ahead of print]. 10.1038/npp.2012.87 22669168 PMC3521995

[B155] Olde Loohuis N. F. Kos A. Martens G. J. Van Bokhoven H. Nadif K. N. Aschrafi A. (2012). MicroRNA networks direct neuronal development and plasticity. Cell Mol. Life Sci. 69, 89–102 10.1007/s00018-011-0788-1 21833581 PMC3249201

[B156] Packer A. N. Xing Y. Harper S. Q. Jones L. Davidson B. L. (2008). The bifunctional microRNA miR-9/MIR-9* regulates REST and co-REST and is downregulated in Huntington’s disease. J. Neurosci. 28, 14341–14346 10.1523/JNEUROSCI.2390-08.2008 19118166 PMC3124002

[B157] Park J. K. Henry J. C. Jiang J. Esau C. Gusev Y. Lerner M. R. Postier R. G. Brackett D. J. Schmittgen T. D. (2011). MiR-132 and miR-212 are increased in pancreatic cancer and target the retinoblastoma tumor suppressor. Biochem. Biophys. Res. Commun. 406, 518–523 10.1016/j.bbrc.2011.02.065 21329664 PMC3069485

[B158] Pinkerton L. E. Hein M. J. Stayner L. T. (2004). Mortality among a cohort of garment workers exposed to formaldehyde: an update. Occup. Environ. Med. 6, 193–200 10.1136/oem.2003.007476 14985513 PMC1740723

[B159] Podlisny M. B. Lee G. Selkoe D. J. (1987). Gene dosage of the amyloid beta precursor protein in Alzheimer’s disease. Science 238, 669–671 10.1126/science.2960019 2960019

[B160] Purdie E. L. Metcalf J. S. Kashmiri S. Codd G. A. (2009). Toxicity of the cyanobacterial neurotoxin β-*N*-methylamino-L-alanine to three aquatic animal species. Amyotroph. Lateral Scler. 2(Suppl. 10), 67–70 10.3109/17482960903273551 19929735

[B161] Rager J. E. Smeester L. Jaspers I. Sexton K. G. Fry R. C. (2011). Epigenetic changes induced by air toxics: formaldehyde exposure alters miRNA expression profiles in human lung cells. Environ. Health Perspect. 119, 494–500 10.1289/ehp.1003323 21147603 PMC3080931

[B162] Ravi A. Gurtan A. M. Kumar M. S. Bhutkar A. Chin C. Lu V. Leese J. A. Jacks T. Sharp P. A. (2012). Proliferation and tumorigenesis of a murine sarcoma cell line in the absence of DICER1. Cancer Cell 21, 848–855 10.1016/j.ccr.2012.04.037 22698408 PMC3385871

[B163] Rivas M. A. Venturutti L. Huang Y. W. Schillaci R. Huang T. H. Elizalde P. V. (2012). Downregulation of the tumor-suppressor miR-16 via progestin-mediated oncogenic signaling contributes to breast cancer development. Breast Cancer Res. 14, R77 10.1186/bcr3187 22583478 PMC3446340

[B164] Robison S. H. Bradley W. G. (1984). DNA damage and chronic neuronal degenerations. J. Neurol. Sci. 64, 11–20 10.1016/0022-510X(84)90051-0 6234379

[B165] Rohn T. T. Rissman R. A. Davis M. C. Kim Y. E. Cotman C. W. Head E. (2002). Caspase-9 activation and caspase cleavage of tau in the Alzheimer’s disease brain. Neurobiol Dis. 11, 341–354 10.1006/nbdi.2002.0549 12505426

[B166] Román G. C. Zhang Z.-X. Ellenberg J. A. (1995). The Neuroepidemiology of Parkinson’s Disease. New York: Marcel Dekker

[B167] Rosenberg D. W. Giardina C. Tanaka T. (2009). Mouse models for the study of colon carcinogenesis. Carcinogenesis 30, 183–196 10.1093/carcin/bgn267 19037092 PMC2639048

[B168] Rovelet-Lecrux A. Hannequin D. Raux G. Le Meur N. Laquerrière A. Vital A. Dumanchin C. Feuillette S. Brice A. Vercelletto M. Dubas F. Frebourg T. Campion D. (2006). APP locus duplication causes autosomal dominant early-onset Alzheimer disease with cerebral amyloid angiopathy. Nat. Genet. 38, 24–26 10.1038/ng1718 16369530

[B169] Rumchev K. B. Spickett J. T. Bulsara M. K. Phillips M. R. Stick S. M. (2002). Domestic exposure to formaldehyde significantly increases the risk of asthma in young children. Eur. Respir. J. 20, 403–408 10.1183/09031936.02.00245002 12212974

[B170] Schaefer A. O’Carroll D. Tan C. L. Hillman D. Sugimori M. Llinas R. Greengard P. (2007). Cerebellar neurodegeneration in the absence of microRNAs. J. Exp. Med. 204, 1553–1558 10.1084/jem.20070823 17606634 PMC2118654

[B171] Schetter A. J. Harris C. C. (2011). Alterations of microRNAs contribute to colon carcinogenesis. Semin. Oncol. 38, 734–742 10.1053/j.seminoncol.2011.08.009 22082759 PMC3217180

[B172] Schmid J. A. Birbach A. (2008). IκB kinase β (IKKβ/IKK2/IKBKB) – a key molecule in signaling to the transcription factor NF-κB. Cytokine Growth Factor Rev. 19, 157–165 10.1016/j.cytogfr.2008.01.006 18308615

[B173] Schouten M. Buijink M. R. Lucassen P. J. Fitzsimmons C. P. (2012). New neurons in aging brains: molecular control by small non-coding RNAs. Front. Neurosci. 6:25 10.3389/fnins.2012.00025 22363255 PMC3281214

[B174] Schratt G. M. Tuebing F. Nigh E. A. Kane C. G. Sabatini M. E. Kiebler M. Greenberg M. E. (2006). A brain-specific microRNA regulates dendritic spine development. Nature 439, 283–289 10.1038/nature04367 16421561

[B175] Sexton K. G. Jeffries H. E. Jang M. Kamens R. M. Doyle M. Voicu I. Jaspers I. (2004). Photochemical products in urban mixtures enhance inflammatory responses in lung cells. Inhalation Toxicol. 16, 107–114 10.1080/08958370490443196 15204799

[B176] Shank R. C. Magee P. N. (1967). Similarities between the biochemical actions of cycasin and dimethylnitrosamine. Biochem. J. 105, 521–527 5583994 10.1042/bj1050521 PMC1198340

[B177] Shen W. B. McDowell K. A. Siebert A. A. Clark S. M. Dugger N. V. Valentino K. M. Jinnah H. A. Sztalryd C. Fishman P. S. Shaw C. A. Jafri M. S. Yarowsky P. J. (2010). Environmental neurotoxin-induced progressive model of parkinsonism in rats. Ann. Neurol. 68, 70–80 10.1002/ana.22018 20582986 PMC2988442

[B178] Shimada M. Langman J. (1970). Repair of the external granular layer of the hamster cerebellum after prenatal and postnatal administration of methylazoxymethanol. Teratology 3, 119–133 10.1002/tera.1420030204 5446879

[B179] Sieber S. M. Correa P. Dalgard D. W. McIntire K. R. Adamson R. H. (1980). Carcinogenicity and hepatotoxicity of cycasin and its aglycone methylazoxymethanol acetate in nonhuman primates. J. Natl. Cancer Inst. 65, 177–189 6248673

[B180] Siegel G. Obernosterer G. Fiore R. Oehman M. Bicker S. Christensen M. Khudayberdiev S. Leuschner P. F. Busch C. J. Kane C. Hübel K. Dekker F. Hedberg C. Rengarajan B. Drepper C. Waldmann H. Kauppinen S. Greenberg M. E. Draguhn A. Rehmsmeier M. Martinez J. Schratt G. M. (2009). A functional screen implicates microRNA-138-dependent regulation of the depalmitoylation enzyme APT1 in dendritic spine morphogenesis. Nat. Cell Biol. 11, 705–716 10.1038/ncb1876 19465924 PMC3595613

[B181] Silber J. R. Blank A. Bobola M. S. Mueller B. A. Kolstoe D. D. Ojemann G. A. Berger M. S. (1996). Lack of the DNA repair enzyme *O*^6^-methylguanine DNA methyltransferase in histologically normal brain adjacent to primary human brain tumors. Proc. Natl. Acad. Sci. U.S.A. 93, 6941–6946 10.1073/pnas.93.14.6941 8692923 PMC38913

[B182] Simón A. M. de Maturana R. L. Ricobaraza A. Escribano L. Schiapparelli L. Cuadrado-Tejedor M. Pérez-Mediavilla A. Avila J. Del Río J. Frechilla D. (2009). Early changes in hippocampal Eph receptors precede the onset of memory decline in mouse models of Alzheimer’s disease. J. Alzheimers Dis. 17, 773–786 19542617 10.3233/JAD-2009-1096

[B183] Simunovic F. Yi M. Wang Y. Macey L. Brown L. T. Krichevsky A. M. Andersen S. L. Stephens R. M. Benes F. M. Sonntag K. C. (2009). Gene expression profiling of substantia nigra dopamine neurons: further insights into Parkinson’s disease pathology. Brain 132, 1795–1809 10.1093/brain/awn323 19052140 PMC2724914

[B184] Singh S. K. (2007). miRNAs: from neurogeneration to neurodegeneration. Pharmacogenomics 8, 971–978 10.2217/14622416.8.8.971 17716230

[B185] Smith P. Y. Delay C. Girard J. Papon M. A. Planel E. Sergeant N. Buée L. Hébert S. S. (2011). MicroRNA-132 loss is associated with tau exon 10 inclusion in progressive supranuclear palsy. Hum. Mol. Genet. 20, 4016–4024 10.1093/hmg/ddr330 21807765

[B186] Sohn O. S. Fiala E. S. Requeijo S. P. Weisburger J. H. Gonzalez F. J. (2001). Differential effects of CYP2E1 status on the metabolic activation of the colon carcinogens azoxymethane and methylazoxymethanol. Cancer Res. 61, 8435–8440 11731424

[B187] Spencer P. S. Ohta M. Palmer V. S. (1987a). Cycad use and motor neurone disease in Kii Peninsula of Japan. Lancet 2, 1462–1463 10.1016/S0140-6736(87)91159-7 2892020

[B188] Spencer P. S. Palmer V. S. Herman A. Asmedi A. (1987b). Cycad use and motor neurone disease in Irian Jaya. Lancet 2, 1273–1274, 1987. 10.1016/S0140-6736(87)91883-6 2890883

[B189] Spencer P. S. Nunn P. B. Hugon J. Ludolph A. C. Ross S. M. Roy D. N. Robertson R. C. (1987c). Guam amyotrophic lateral sclerosis-parkinsonism-dementia linked to a plant excitant neurotoxin. Science 237, 517–522 10.1126/science.3603037 3603037

[B190] Spencer P. S. Palmer V. S. Ludolph A. C. (2005). On the decline and etiology of high-incidence motor system disease in West Papua (southwest New Guinea). Mov. Disord. 20(Suppl 12), S119–S126 10.1002/mds.20322 16092101

[B191] Staropoli J. F. (2008). Tumorigenesis and neurodegeneration: two sides of the same coin? Bioessays 30, 719–727 10.1002/bies.20784 18623069

[B192] Sumiyoshi K. Kubota S. Ohgawara T. Kawata K. Nishida T. Shimo T. Yamashiro T. Takigawa M. (2010). Identification of miR-1 as a micro RNA that supports late-stage differentiation of growth cartilage cells. Biochem. Biophys. Res. Commun. 402, 286–290 10.1016/j.bbrc.2010.10.016 20937250

[B193] Suzuki H. Takatsuka S. Akashi H. Yamamoto E. Nojima M. Maruyama R. Kai M. Yamano H. O. Sasaki Y. Tokino T. Shinomura Y. Imai K. Toyota M. (2011). Genome-wide profiling of chromatin signatures reveals epigenetic regulation of microRNA genes in colorectal cancer. Cancer Res. 71, 5646–5658 10.1158/1538-7445.AM2011-1147 21734013

[B194] Takigawa Y. Brown A. M. (2008). signaling in liver cancer. Curr. Drug Targets 9, 1013–1024 10.2174/138945008786786127 18991612 PMC4446985

[B195] Takihashi M. Wakabayashi K. (2004). Gene mutations and altered gene expression in azoxymethane-induced colon carcinogenesis in rodents. Cancer Sci. 95, 475–480 10.1111/j.1349-7006.2004.tb03235.x 15182426 PMC11158569

[B196] Tanganelli S. Antonelli T. Tomasini M. C. Beggiato S. Fuxe K. Ferraro L. (2012). Relevance of dopamine D(2)/neurotensin NTS1 and NMDA/neurotensin NTS1 receptor interaction in psychiatric and neurodegenerative disorders. Curr. Med. Chem. 19, 304–316 22335510 10.2174/092986712803414268

[B197] Theuns J. Marjaux E. Vandenbulcke M. Van Laere K. Kumar-Singh S. Bormans G. Brouwers N. Van den Broeck M. Vennekens K. Corsmit E. Cruts M. De Strooper B. Van Broeckhoven C. Vandenberghe R. (2006). Alzheimer dementia caused by a novel mutation located in the APP C-terminal intracytosolic fragment. Hum. Mutat. 27, 888–896 10.1002/humu.20402 16917905

[B198] Thorgeirsson U. P. Dalgard D. W. Reeves J. Adamson R. H. (1994). Tumor incidence in a chemical carcinogenesis study of nonhuman primates. Regul. Toxicol. Pharmacol. 19, 130–151 10.1006/rtph.1994.1013 8041912

[B199] Tong Z. Zhang J. Luo W. Wang W. Li F. Luo H. Lu J. Zhou J. Wan Y. He R. (2011). Urine formaldehyde level is inversely correlated to mini mental state examination scores in senile dementia. Neurobiol. Aging 32, 31–41 10.1016/j.neurobiolaging.2009.07.013 19879019

[B200] Tremblay M. E. Riad M. Bouvier D. Murai K. K. Pasquale E. B. Descarries L. Doucet G. (2007). Localization of EphA4 in axonal terminals and dendritic spines of adult rat hippocampus. J. Comp. Neurol. 501, 691–702 10.1002/cne.21263 17299751

[B201] Tufekci K. U. Meuwissen R. Genc S. Genc K. (2012). Inflammation in Parkinson’s disease. Adv. Protein Chem. Struct. Biol. 88, 69–132 10.1016/B978-0-12-398314-5.00004-0 22814707

[B202] Tuthill R. W. (1984). Woodstoves, formaldehyde, and respiratory disease. Am. J. Epidemiol. 120, 952–955 6507432 10.1093/oxfordjournals.aje.a113966

[B203] Vaughan T. L. Stewart P. A. Teschke K. Lynch C. F. Swanson G. M. Lyon J. L. Berwick M. (2000). Occupational exposure to formaldehyde and wood dust and nasopharyngeal carcinoma. Occup. Environ. Med. 57, 376–384 10.1136/oem.57.6.376 10810126 PMC1739963

[B204] Veeriah S. Morris L. Solit D. Chan T. A. (2010). The familial Parkinson disease gene *PARK2* is a multisite tumor suppressor on chromosome 6q25.2–27 that regulates cyclin E. Cell Cycle 9, 1451–1452 10.4161/cc.9.8.11583 20372088 PMC2921461

[B205] Villa C. Fenoglio C. De Riz M. Clerici F. Marcone A. Benussi L. Ghidoni R. Gallone S. Cortini F. Serpente M. Cantoni C. Fumagalli G. Martinelli Boneschi F. Cappa S. Binetti G. Franceschi M. Rainero I. Giordana M. T. Mariani C. Bresolin N. Scarpini E. Galimberti D. (2011). Role of hnRNP-A1 and miR-590-3p in neuronal death: genetics and expression analysis in patients with Alzheimer disease and frontotemporal lobar degeneration. Rejuvenation Res 14, 275–281 10.1089/rej.2010.1123 21548758

[B206] Vo N. Klein M. E. Varlamova O. Keller D. M. Yamamoto T. Goodman R. H. Imprey S. (2005). A cAMP-response element binding protein-induced microRNA regulates neuronal morphogenesis. Proc. Natl. Acad. Sci. U.S.A. 102, 16426–16431 10.1073/pnas.0508448102 16260724 PMC1283476

[B207] Wan G. Mathur R. Hu X. Zhang X. Lu X. (2011). miRNA response to DNA damage. Trends Biochem. Sci. 36, 478–484 10.1016/j.tibs.2011.06.002 21741842 PMC3532742

[B208] Wang B. Hsu S. H. Majumder S. Kutay H. Huang W. Jacob S. T. Ghoshal K. (2010). TGFbeta-mediated upregulation of hepatic miR-181b promotes hepatocarcinogenesis by targeting TIMP3. Oncogene 29, 1787–1797 10.1038/onc.2010.247 20023698 PMC2845743

[B209] Wang M. Gu H. Wang S. Qian H. Zhu W. Zhang L. Zhai C. Tao Y. Xu W. (2012). Circulating miR-17-5p and miR-20a: molecular markers for gastric cancer. Mol. Med. Report 5, 1514–1520 22406928 10.3892/mmr.2012.828

[B210] Wang X. (2008). miRDB: a microRNA target prediction and functional annotation database with a wiki interface, RNA 14, 1012–1017 10.1261/rna.1252608 18426918 PMC2390791

[B211] Wang X. El Naqa I. M. (2008). Prediction of both conserved and nonconserved microRNA targets in animals. Bioinformatics 24, 325–332 10.1093/bioinformatics/btn132 18048393

[B212] Wei Q. Li Y. X. Liu M. Li X. Tang H. (2012). MiR-17-5-p targets TP53INP1 and regulates cell proliferation and apoptosis of cervical cancer cells. IUBMB Life 64, 697–704 10.1002/iub.1051 22730212

[B213] Weisskopf M. G. Gallo V. O’Reilly E. J. Vineis P. Ascherio A. (2010). Smoking may be considered an established risk factor for sporadic ALS. Neurology 74, 1927–1928 10.1212/WNL.0b013e3181e038e9 20549870

[B214] Weisskopf M. G. McCullough M. L. Morozova N. Calle E. E. Thun M. J. Ascherio A. (2005). Prospective study of occupation and amyotrophic lateral. Am. J. Epidemiol. 162, 1146–1152 10.1093/aje/kwi343 16269579

[B215] Wheeler G. Ntounia-Fousara S. Granda B. Rathjen T. Dalmay T. (2006). Identification of new central nervous system specific mouse microRNAs. FEBS Lett. 580, 2195–2200 10.1016/j.febslet.2006.03.019 16566924

[B216] Whiting M. G. (1988). “Toxicity of cycads: implications for neurodegenerative diseases and cancer,” in Transcripts of Four Cycad Conferences (New York: Third World Medical Research Foundation), 1–294 Available at: http://www.ohsu.edu/xd/education/continuing-education/global-health-center/gh-research/upload/Cycad_Book_IA.pdf; 294–458. Available at: http://www.ohsu.edu/xd/education/continuing-education/global-health-center/gh-research/upload/Cycad_Book_IB.pdf

[B217] Wieslander G. Norbäck D. Björnsson E. Janson C. Boman G. (1997). Asthma and the indoor environment: the significance of emission of formaldehyde and volatile organic compounds from newly painted indoor surfaces. Int. Arch. Occup. Environ. Health 69, 115–124 10.1007/s004200050125 9001918

[B218] Wu W. K. Law P. T. Lee C. W. Cho C. H. Fan D. Wu K. Yu J. Sung J. J. (2011). MicroRNAs in colorectal cancer: from benchtop to bedside. Carcinogenesis 32, 247–253 10.1093/carcin/bgq287 21081475

[B219] Yamamoto Y. Yoshioka Y. Minoura K. Takashashi R. U. Takeshita F. Taya T. Horii R. Fukuoka Y. Kato T. Kosaka N. Ochiyra T. (2011). An integrative genomic analysis revealed the relevance of microRNA and gene expression for drug-resistance in human breast cancer cells. Mol. Cancer 10, 135 10.1158/1535-7163.TARG-11-A135 22051041 PMC3247093

[B220] Yanaihara N. Caplen N. Bowman E. Seike M. Kumamoto K. Yi M. Stephens R. M. Okamoto A. Yokota J. Tanaka T. Calin G. A. Liu C. G. Croce C. M. Harris C. C. (2006). Unique microRNA molecular profiles in lung cancer diagnosis and prognosis. Cancer Cell 9, 189–198 10.1016/j.ccr.2006.01.025 16530703

[B221] Yoo A. S. Staahl B. T. Chen L. Crabtree G. R. (2009). MicroRNA-mediated switching of chromatin-remodelling complexes in neural development. Nature 460, 642–646 19561591 10.1038/nature08139 PMC2921580

[B222] Yu J. Ohuchida K. Mizumoto K. Fujita H. Nakata K. Tanaka M. (2010). MicroRNA miR-17-5p is overexpressed in pancreatic cancer, associated with a poor prognosis, and involved in cancer cell proliferation and invasion. Cancer Bio. Ther. 10, 748–757 10.4161/cbt.10.8.13083 20703102

[B223] Yu J. Y. Chung K. H. Deo M. Thompson R. C. Turner D. L. (2008). MicroRNA miR-124 regulates neurite outgrowth during neuronal differentiation. Exp. Cell Res. 314, 1618–1633 10.1016/j.yexcr.2008.08.001 PMC2702206 18619591

[B224] Zhang L. Freeman L. E. Nakamura J. Hecht S. S. Vandenberg J. J. Smith M. T. Snawane B. R. (2010a). Formaldehyde and leukemia: epidemiology, potential mechanisms, and implications for risk assessment. Environ. Mol. Mutagen. 51, 181–191 19790261 10.1002/em.20534 PMC2839060

[B225] Zhang L. Tang X. Rothman N. Vermeulen R. Ji Z. Shen M. Qiu C. Guo W. Liu S. Reiss B. Freeman L. B. Ge Y. Hubbard A. E. Hua M. Blair A. Galvan N. Ruan X. Alter B. P. Xin K. X. Li S. Moore L. E. Kim S. Xie Y. Hayes R. B. Azuma M. Hauptmann M. Xiong J. Stewart P. Li L. Rappaport S. M. Huang H. Fraumeni J. F. Jr. Smith M. T. Lan Q. (2010b). Occupational exposure to formaldehyde, hematotoxicity, and leukemia-specific chromosome changes in cultured myeloid progenitor cells. Cancer Epidemiol. Biomarkers Prev. 19, 80–88 10.1158/1055-9965.DISP-10-A80 20056626 PMC2974570

[B226] Zhang H. Qi M. Li S. Qi T. Mei H. Huang K. Zheng L. Tong Q. (2012a). microRNA-9 targets matrix metalloproteinase 14 to inhibit invasion, metastasis, and angiogenesis of neuroblastoma cells. Mol. Cancer Ther. 11, 1454–1466 10.1158/1535-7163.MCT-11-0548 22564723

[B227] Zhang W. Zhang J. Hoadley K. Kushwaha D. Ramakrishnan V. Li S. Kang C. You Y. Jiang C. Song S. W. Jiang T. Chen C. C. (2012b). miR-181d: a predictive glioblastoma biomarker that downregulates MGMT expression. Neuroloncology 14, 712–719 10.1093/neuonc/nos089 PMC3367855 22570426

[B228] Zhang X. Wang H. Zhang S. Song J. Zhang Y. Wei X. Feng Z. (2012c). MiR-134 functions as a regulator of cell proliferation, apoptosis, and migration involving lung septation. In vitro Cell. Dev. Biol. Anim. 48, 131–136 10.1007/s11626-012-9482-3 22259016

[B229] Zhang S. Hao J. Xie F. Hu X. Liu C. Tong J. Zhou J. Wu J. Shao C. (2011). Downregulation of miR-132 by promoter methylation contributes to pancreatic cancer development. Carcinogenesis 32, 1183–1189 10.1093/carcin/bgq286 21665894

[B230] Zhang Z. X. Anderson D. W. Mantel N. Román G. C. (1996). Motor neuron disease on Guam: geographic and familial occurrence, 1956–1985. Acta Neurol. Scand. 94, 51–59 10.1111/j.1600-0404.1996.tb00039.x 8874594

[B231] Zhao S. Dou W. He L. Liang S. Tie J. Liu C. Li T. Lu Y. Mo P. Shi Y. Wu K. Nie Y. Fan D. (2012). MicroRNA-7 functions as an anti-metastatic microRNA in gastric cancer by targeting insulin-like growth factor-1 receptor. Oncogene. [Epub ahead of print]. 10.1038/onc.2012.156 22614005

[B232] Zhou X. Zhao F. Wang Z. N. Song Y. X. Chang H. Chiang Y. Xu H. M. (2012). Altered expression of miR-152 and miR-148a in ovarian cancer is related to cell proliferation. Oncol. Rep. 27, 447–454 21971665 10.3892/or.2011.1482

[B233] Zhu H. Dougherty U. Robinson V. Mustafi R. Pekow J. Kupfer S. Li Y.-C. Hart J. Goss K. Fichera A. Joseph L. Bissonnette M. (2011). EGFR signals downregulate tumor suppressors miR-143 and miR-145 in western diet–promoted murine colon cancer: role of G1 regulators. Mol. Cancer Res. 9, 960–975 10.1158/1541-7786.MCR-10-0531 21653642 PMC3819602

